# Oral Motion‐Powered Smart Dental Implant Abutment for In Situ Antibacterial and Cell Adhesion Through Piezoelectric Effect

**DOI:** 10.1002/advs.202523944

**Published:** 2026-05-26

**Authors:** Xiyu Shi, Xiaoyu Han, Yanhui Lu, Yuan Chai, Baiyan Xiao, Shuo Liu, Boon Chin Heng, Tingting Wu, Tingjun Li, Qiaomei Ren, Ting Song, Le Chen, Dong Han, Yaru Guo, Xuliang Deng, Xuehui Zhang

**Affiliations:** ^1^ Department of Dental Materials & Dental Medical Devices Testing Center Peking University School and Hospital of Stomatology Beijing P. R. China; ^2^ Department of Prosthodontics Peking University School and Hospital of Stomatology Beijing P. R. China; ^3^ Department of Geriatric Dentistry Peking University School and Hospital of Stomatology Beijing P. R. China; ^4^ National Center for Stomatology National Clinical Research Center for Oral Diseases National Engineering Research Center of Oral Biomaterials and Digital Medical Devices NMPA Center for Innovation and Research in Regulatory Science Beijing Laboratory of Biomedical Materials & Beijing Key Laboratory of Biomaterials for Oral Disease NHC Key Laboratory of Digital Stomatology Peking University School and Hospital of Stomatology Beijing P. R. China; ^5^ Oral Translational Medicine Research Center Joint Training base For Shanxi Provincial Key Laboratory in Oral and Maxillofacial Repair Reconstruction and Regeneration The First People's Hospital of Jinzhong Jinzhong P. R. China; ^6^ Peking University Hospital of Stomatology Sanya Division (Sanya Stomatology Center) Sanya P. R. China

**Keywords:** antibacterial effect, cell adhesion, dental implant abutments, oral motion, piezoelectric effects

## Abstract

The long‐term clinical success of dental implants is critically dependent on achieving stable soft‐tissue integration while preventing bacterial colonization and subsequent peri‐implantitis. Piezoelectric biomaterials offer a route to address this challenge, yet the potential to harness ambient oral motions (e.g., mastication) as a continuous power source for autonomous therapeutic action remains largely unexplored. Here, we report a motion‐activated smart dental implant abutment (SDIA) constructed from a toughened piezoelectric composite comprising a 3D interconnected barium titanate (BaTiO_3_) ceramic framework infiltrated with a high‐strength polymer matrix. This architecture imparts exceptional flexural strength and fracture toughness via polymer‐mediated crack deflection. Under simulated oral pressure, the SDIA demonstrates efficient biomechanical‐to‐electrical energy conversion, driving a potent piezo‐catalytic effect that generates sufficient reactive oxygen species (ROS) to eradicate 96.5% of *E. coli* and 89.7% of *S. aureus*, and robustly inhibits biofilm formation. Concurrently, the motion‐induced electrical cues directly modulate fibroblast behavior by upregulating the MAPK and PI3K‐Akt signaling pathways, substantially enhancing cell adhesion and proliferation. This work establishes a new paradigm for smart biomaterials, demonstrating that harnessing natural physiological motion can power autonomous implants capable of delivering synergistic antibacterial and regenerative therapies to prevent clinical device failure.

## Graphical abstract

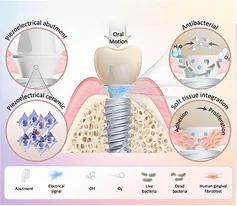

## 1 Introduction

Dental implants are increasingly being used for the restoration of missing teeth due to their aesthetic appeal and ability to restore occlusal function [1, 2]. Stable soft tissue integration between peri‐implant soft tissues and antibacterial properties are both essential for attenuating pathogen penetration, preventing peri‐implantitis, and maintaining long‐term implant stability [3, 4, 5, 6, 7]. The surface modification strategies of abutments have been developed to enhance peri‐implant cell adhesion and prevent bacterial infection. Bioactive molecular coatings, such as sericin@Cu coating, collagen, and specific peptide sequences, have been utilized to improve fibroblast attachment and soft tissue sealing [8, 9, 10]. Antibiotics, silver nanoparticles, and antimicrobial peptides have been developed for the preparation of abutment coatings to inhibit bacterial colonization and prevent the formation of biofilms [11, 12, 13]. However, their clinical effects are still compromised by unsatisfactory integration, suboptimal antimicrobial efficacy, and potential toxicity to adjacent tissues [14, 15, 16, 17]. Hence, there is a dire need to develop novel implant abutments for sustainable implant performance.

Many piezoelectric materials, such as barium titanate (BTO) [18, 19], potassium sodium niobate (KNN) [20], and PVDF [21], have been developed to enhance cell adhesion [22] and prevent bacterial infection based on the electrical microenvironment or generation of piezoelectric charges [23]. For example, a bionic conductive integrated peri‐implant gingiva has been developed based on flexible piezoelectric films and conductive polymer networks, which have antibacterial and cell adhesion functions under ultrasonic stimulation [24]. Moreover, multifunctional piezoelectric systems, such as shape‐adaptive adhesive patches [25] and injectable anti‐adhesion hydrogels [26], have been designed to promote tissue regeneration, inhibit bacterial infection, and reduce inflammation by providing localized electrical stimulation and physical protection at injury sites. Hence, biophysical stimulation approaches based on piezoelectric and piezo‐catalysis effects might be promising alternative strategies for the design of such functional implant abutments. However, due to the poor mechanical durability and insufficient piezoelectric activity of most piezoelectric materials [27, 28], the development of piezoelectric material‐based implant abutment with optimal mechanical properties, inherent antimicrobial activity, and cell adhesion capacity under physiological oral conditions remains a significant challenge in dental biomaterial engineering.

**SCHEME 1:**
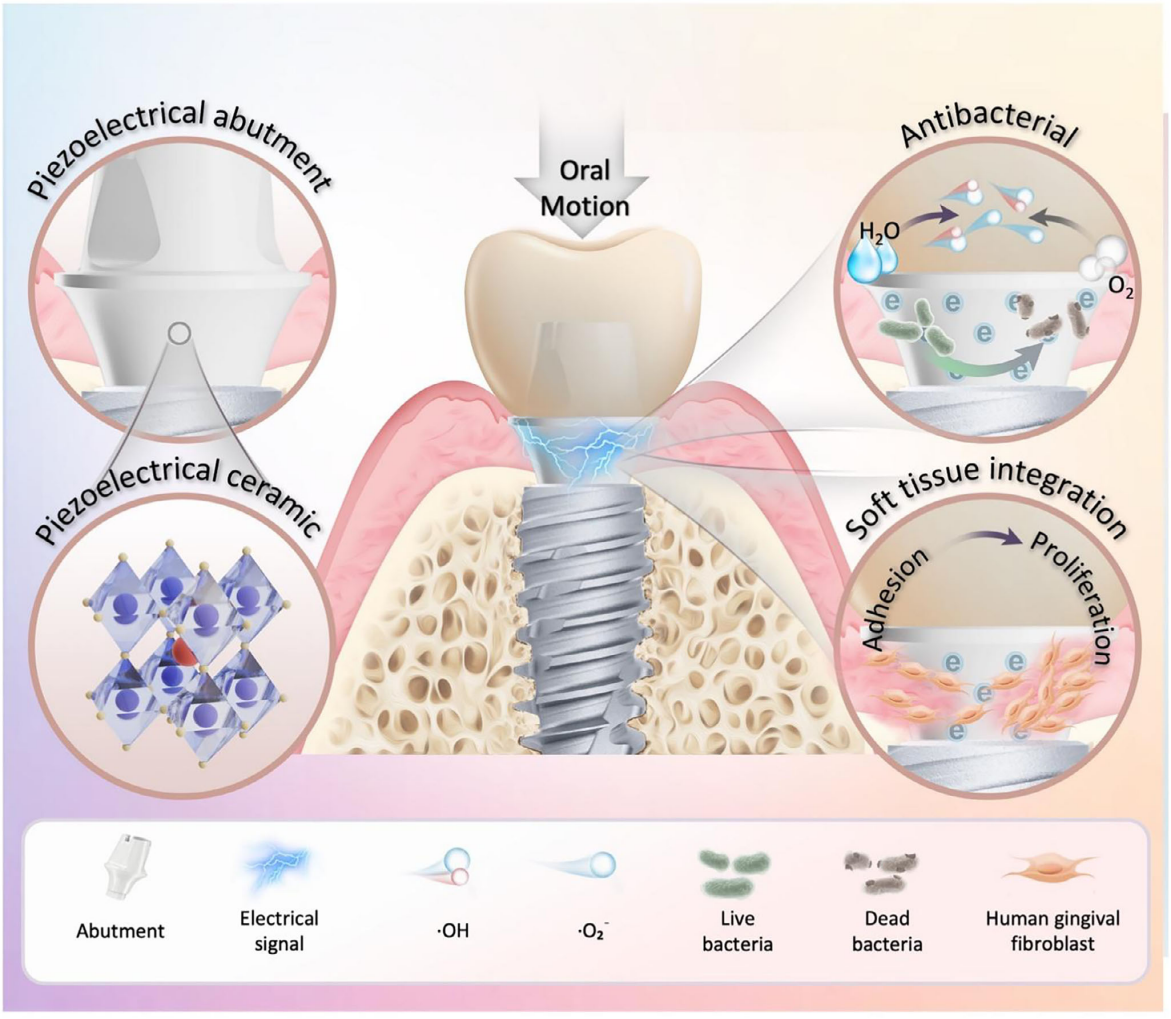
Schematic diagram of the oral motion‐powered smart dental implant abutments in the dental field. We proposed a method based on the piezoelectric effect and fabricated an implant abutment on the basis of piezoelectric ceramic. Under physiological occlusal pressure, the oral motion‐powered smart dental implant abutments interacted with water and oxygen, promoting the formation of reactive oxygen species and achieving a synergistic and highly efficient piezoelectric dynamic killing effect on *Staphylococcus aureus* (*S. aureus*) and *Escherichia coli* (*E. coli*), thereby further inhibiting the formation of biofilms. In addition, physiological occlusal electrical stimulation can enhance the adhesion and proliferation of fibroblasts.

Here, we developed an oral motion‐powered smart dental implant abutment (SDIA) based on prevalent intermittent pressure stimulus of teeth when chewing, formed by a resin‐reinforced and toughened piezoelectric BTO ceramic composite material system (BTO‐R), which displayed enhanced antibacterial and cell adhesion properties (Scheme 1). Specifically, vacuum‐assisted resin permeation technology was utilized to construct BTO‐R, which displayed excellent mechanical properties and piezoelectric response. By combining the piezoelectric effect with oral motion, SDIA excited the piezoelectric charge of the abutment, achieving reactive oxygen species (ROS)‐mediated antibacterial effects. Moreover, SDIA also promoted soft tissue cell adhesion by regulating the MAPK and PI3K‐Akt signaling pathways. Compared to existing implant abutments that function to promote soft tissue healing and resist infection, this study achieved synergy between the piezoelectric effect and occlusal movement, thereby breaking through the technical bottleneck in the design of intrinsically infection‐resistant abutments. Hence, we successfully constructed a self‐antibacterial abutment material system for implant restoration, establishing a new material design paradigm for effectively preventing peri‐implantitis and enhancing the long‐term stability of implant restoration.

## 2 Results and Discussion

### 2.1 Design, Preparation, and Characterization of SDIA

**FIGURE 1:**
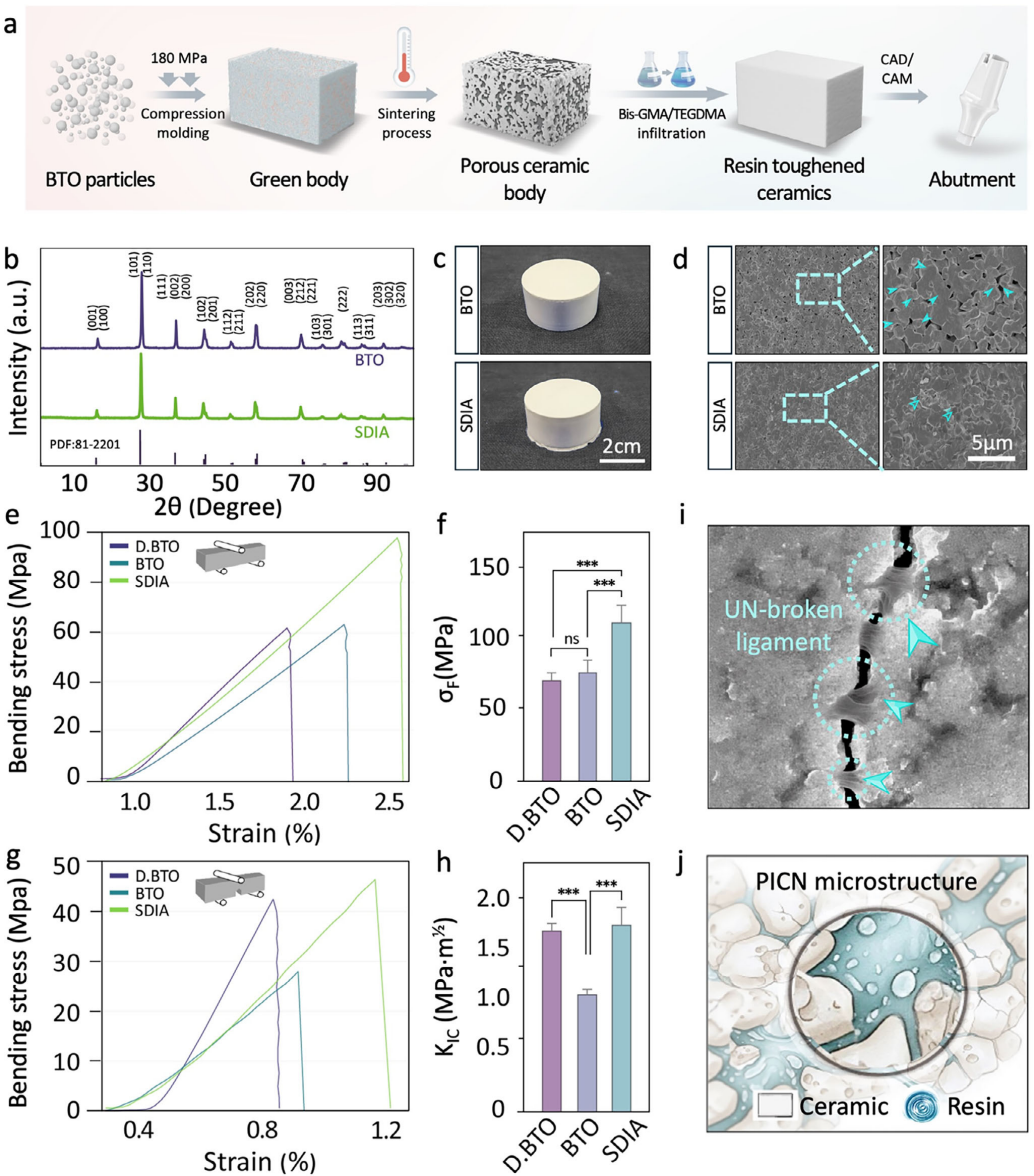
Fabrication strategy and mechanical characterization of resin‐infiltrated BaTiO_3_ Smart Dental Implant Abutment (SDIA). (a) Schematic of the polymer‐infiltrated‐ceramic‐network (PICN) fabrication strategy utilizing vacuum‐assisted resin permeation technology to create reinforced and toughened BaTiO_3_ composite materials. (b) X‐ray diffraction (XRD) patterns confirm the retention of the perovskite crystal phase post‐sintering and resin infiltration, demonstrating preservation of piezoelectric properties during material preparation. (c) Macroscopic appearance comparing pure BaTiO_3_ (BTO) ceramic precursors with SDIA specimens after resin infiltration across various sintering temperatures (1000–1300°C). (d) Scanning electron microscopy (SEM) micrographs show resin infiltration within the through‐pores, with porosity decreasing progressively as sintering temperature increases from 1000°C to 1300°C. (e, f) Flexural strength (σ_f) and (g, h) Single‐edge V‐notch beam (SEVNB) fracture toughness (K_IC) measured per ISO 6872 standard. SDIA with 1200°C preform achieves optimal mechanical properties (σ_f ≈ 100 MPa, K_IC ≈ 1.5‐1.6 MPa·m ^−2^). (i) Crack‐tip scanning electron micrographs reveal polymer bridging and pull‐out mechanisms at the crack interface. (j) Schematic illustration of the polymer bridging toughening mechanism. N = 5 specimens per group, mean ± SD. Ns, not significant; ^*^ *p* < 0.05; ^**^ *p* < 0.01; ^***^ *p* < 0.001. One‐way ANOVA with Tukey's post‐hoc test.

BTO materials are typically applied in the form of ultrathin films in the past due to their poor mechanical durability, which limited their application in the field of biomaterial prosthetics [21, 22]. In order to optimize the mechanical properties of BTO, we designed reinforced and toughened BTO‐R utilizing polymer‐infiltrated‐ceramic‐network (PICN) structure technology, and used these to fabricate the SDIA by cutting (Figure 1a). PICN structure is composed primarily of a porous ceramic structure with an interpenetrating resin phase. Materials with PICN structure possess the hardness, strength, and wear resistance of ceramics, as well as the toughness of resins [29, 30]. We shaped BTO powder through cold isostatic pressing, and then sintered it into a ceramic matrix. By optimizing the sintering temperature from 1000–1300°C, a graded series of BTO ceramic precursors with dynamically‐tuned porosity were fabricated. The results of crystal phase stability (XRD) tests showed that the crystals of BTO after sintering at different temperatures was similar to that of the ordinary BTO. This indicated that the material preparation process did not change the piezoelectric properties of the material itself (Figure 1b and Figure S1). The color of BTO ceramic precursors deepened as the sintering temperature rose. Meanwhile, the volume of the material also shrank as the sintering temperature rose (Figure S2). Figure S3 showed that the porosity gradually decreased as the sintering temperature increased. The ceramic precursors had the highest porosity, reaching 38.3% sintered at 1000°C, while the porosity of the ceramic matrix was close to 0% sintered at 1300°C (Figure S3). This result was also confirmed by scanning electron microscopy (SEM) (Figure S4).

To determine the optimal resin composition ratio, we carried out vacuum resin toughening and thermal curing treatment of BTO ceramic precursors prepared at different sintering temperatures. Comparing the morphology of the BTO ceramic precursors, resin toughening did not affect the color of SDIA (Figure 1c and Figure S5). The SEM results showed that the resin filled the pores of the BTO ceramic precursor, and the resin content also decreased with an increase in sintering temperature (Figure 1d and Figure S6). Further mechanical analysis in accordance with the ISO 6872 standard revealed that the flexural strength of SDIA increased to 100 MPa and the fracture toughness reached 1.5‐1.6 MPa·m^−2^ when the BTO ceramic precursor was sintered at 1200°C (Figure 1e–h and Figure S7). To further explore the optimization mechanism for improving the mechanical properties of SDIA, SEM showed that the crack bridging phenomenon of organic polymer materials existed at the crack of SDIA (Figure 1i,j), thereby clarifying that the organic polymer materials enhanced the flexural strength and fracture toughness of BTO through crack bridging [31, 32]. These results thus confirmed that a process for preparing piezoelectric resin composite abutments with fine mechanical properties was successfully established.

### 2.2 The Piezoelectric Effect of SDIA

**FIGURE 2:**
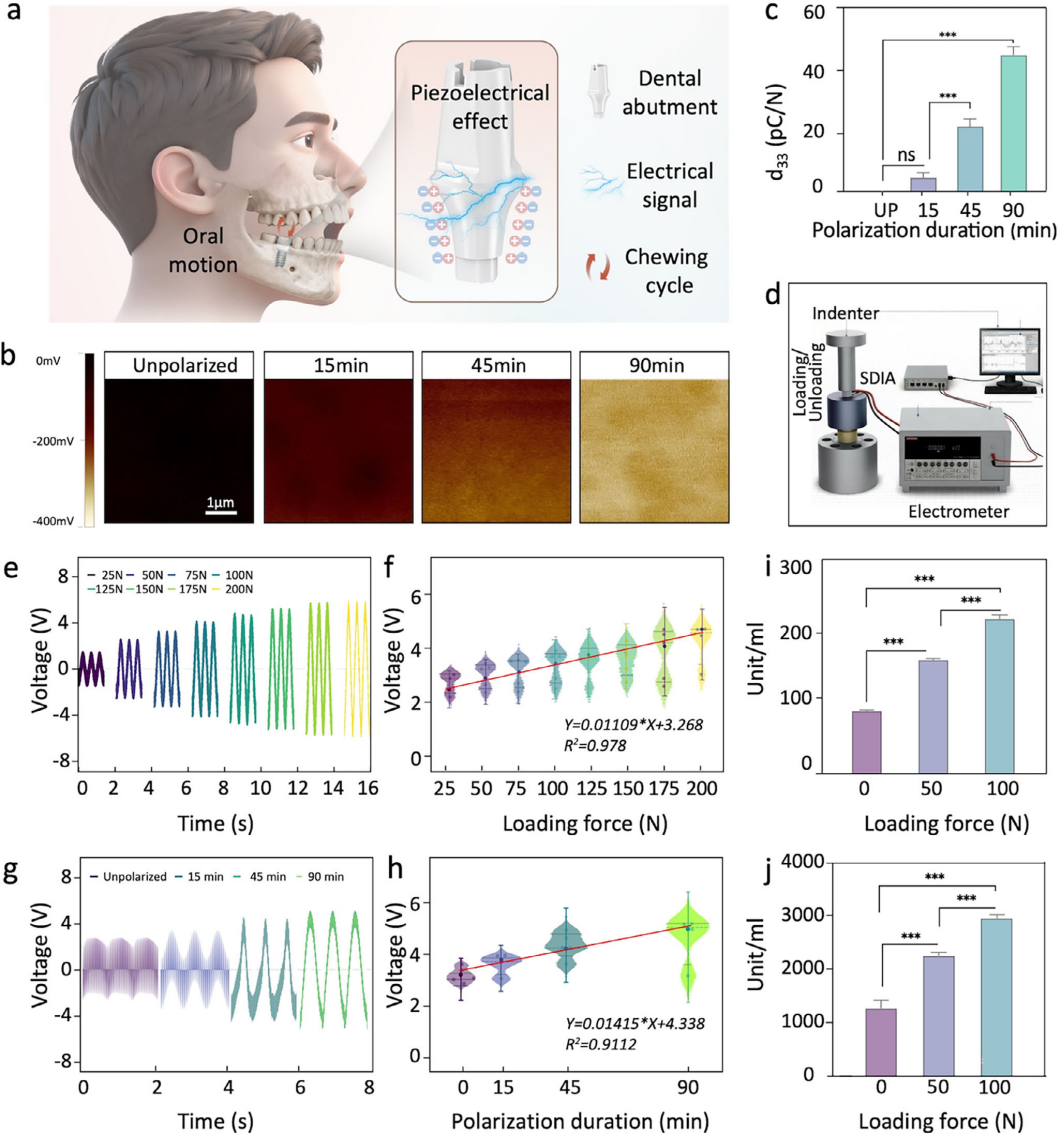
SDIA composite materials demonstrating tunable electrical output and catalytic reactive oxygen species (ROS) generation under physiological chewing forces. (a) Schematic diagram of the electrical signal generated by the piezoelectric abutment under biting force. (b, c) Corona poling for 0–90 min programs the polarization state of SDIA, yielding time‐dependent increases in the piezoelectric constant (d*_33_)* and surface potential measured by Kelvin probe force microscopy. (d) Schematic diagram of the customized chewing‐force emulator system applying cyclic compression at 1.5 Hz frequency with forces ranging from 0–200 N, while simultaneously recording open‐circuit piezoelectric voltage output. (e, f) At fixed polarization duration (90 min), piezoelectric voltage output increases with applied force amplitude. (g, h) At fixed applied force (200N), piezoelectric voltage scales approximately linearly with poling time, with marginal additional gains beyond 90 min of poling. (i, j) UV‐Vis microplate assays quantify hydroxyl radicals (•OH) and superoxide anions (•O_2_^−^), demonstrating that reactive oxygen species (ROS) generation increases dose‐dependently with mechanical force and polarization history, establishing a two‐dimensional programmable catalytic window. n = 3, mean ± SD. Ns, not significant; ^*^ *p* < 0.05; ^**^ *p* < 0.01; ^***^ *p* < 0.001. One‐way ANOVA with Tukey's or Kruskal‐Wallis test. Linear regression slopes are displayed with 95% confidence intervals (CI) and R^2^ values.

SDIA exhibits typical piezoelectric characteristics when subjected to oral motion pressure (Figure 2a). In order to achieve optimal biological effects of SDIA in clinical situations, it is necessary to establish an effective method for adjusting the piezoelectric intensity, as well as elucidate the specific piezoelectric intensity that yields the best biological effect. As a piezoelectric material, the electromechanical response of BTO can be enhanced by polarization treatment [33, 34]. Based on this principle, we chose to adjust the electromechanical response of SDIA through the optimization of polarization duration. The SDIAs were polarized for varying durations of 0, 15, 45, and 90 min, respectively, and the piezoelectric constant (*d* ***_33_***) and surface potential test data indicate that the electromechanical response intensified with increasing polarization time duration (Figure 2b,c). This finding thus demonstrated that the electromechanical response performance of SDIA can be modulated by controlling the polarization time.

To measure the actual piezoelectric intensity, we utilized a fatigue tester to simulate the oral biting movements, applying varying levels of pressure to the material and measuring the piezoelectric voltage using an electrometer (Figure 2d). We applied pressure at a frequency of 1.5 Hz, which is the general frequency of oral motion [35], with a force range of 0–200 N, which is the typical biting force range that posterior dental implants are regularly exposed to [36]. As expected, the piezoelectric strength increased with an extension of polarization time duration, exhibiting a linear relationship (Figure 2e,f). Additionally, it is noteworthy that the piezoelectric strength of samples showed no significant increase after 90 min (Figure S8), suggesting that with the current equipment conditions, the maximum polarization strength was achieved after 90 min of polarization. Furthermore, under the same polarization time for the samples, the piezoelectric strength also increased with an increase in pressure values, exhibiting a linear relationship (Figure 2g,h). Both ·OH and ·O_2_^−^quantification, performed by UV–vis spectrophotometry, confirmed ROS production under the tested conditions, with the production trend of both species exhibiting consistent patterns across replicates. Furthermore, the ·OH and ·O_2_^−^ levels increased progressively with the applied mechanical force, indicating that ROS generation could be modulated by loading intensity (Figure 2i,j). These results thus suggested that the piezoelectric intensity of SDIA can be dynamically regulated by the bite force and polarization intensity, with power supply being adjusted according to demand, based on the functional requirements.

### 2.3 SDIAs Demonstrated Excellent Biocompatibility and Promoted the Adhesion of Soft Tissue Integration‐Related Cells In Vitro

**FIGURE 3:**
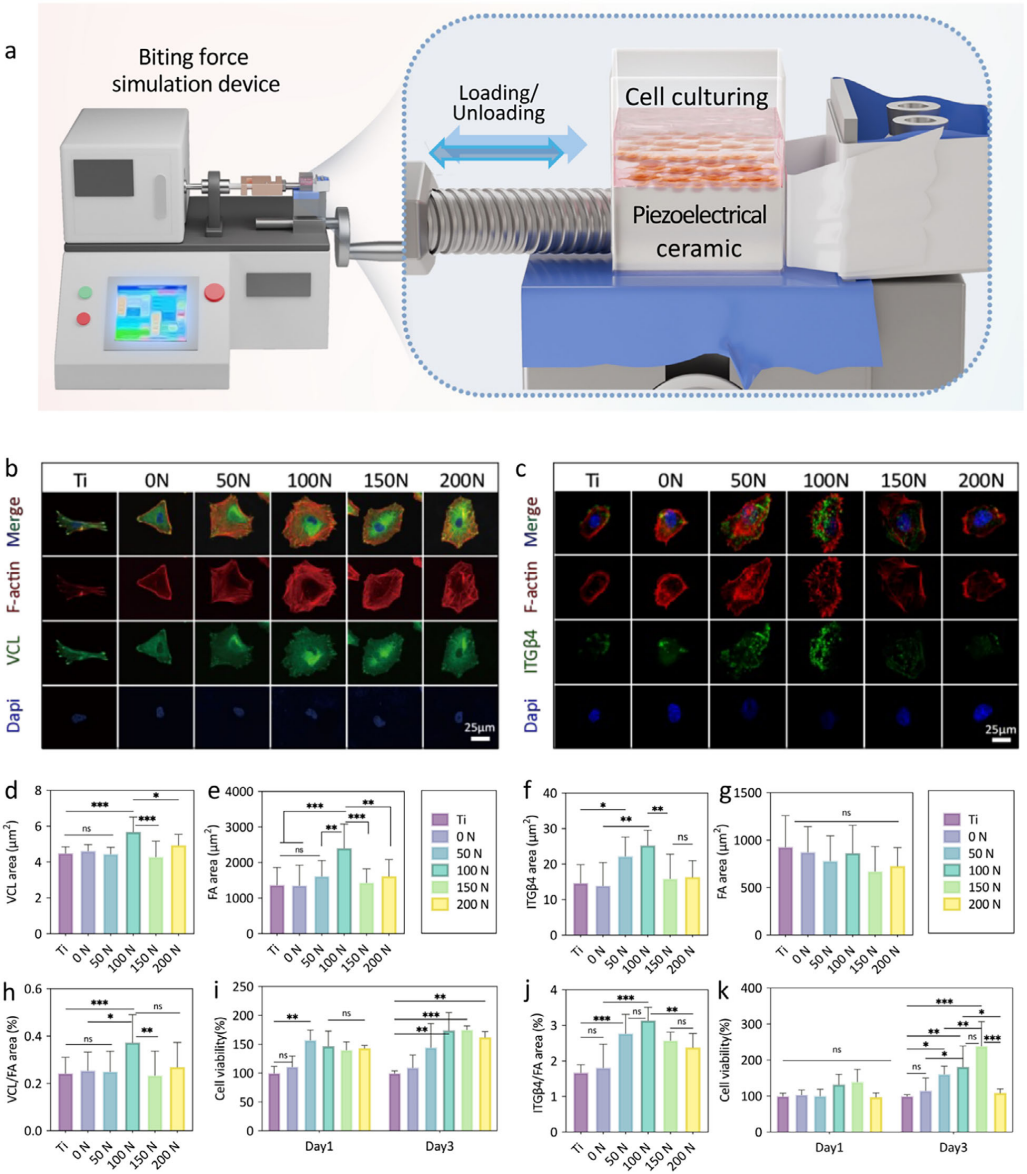
Piezoelectric stimulation enhances HGFs and HOKs adhesion, cytoskeletal organization, and proliferation. (a) Schematic illustration of the customized lateral compression apparatus designed to simulate physiologically relevant oral chewing forces while generating controlled piezoelectric charge under vertical stress conditions. (b) Representative laser‐scanning confocal immunofluorescence images of human gingival fibroblasts (HGFs) stained for filamentous actin (F‐actin, phalloidin, red), focal adhesion marker vinculin (VCL, green), and nuclei (DAPI, blue) under increasing piezoelectric voltage stimulation (0 N, 50 N, 100 N). (d, e, h) Quantification of cytoskeletal organization and adhesion metrics: (d) F‐actin coverage area, (e) vinculin‐positive focal adhesion (VCL) area, and h) the ratio of VCL area to F‐actin area, showing peak adhesion at 4.45 V (100 N). (i) Cell viability assay (CCK‐8) on Days 1 and 3 demonstrates significantly higher metabolic activity on SDIA compared to titanium (Ti) control. (c) Representative immunofluorescence images of human oral keratinocytes (HOKs) stained for F‐actin (phalloidin, red), hemidesmosome marker integrin α4 (ITG4, green), and nuclei (DAPI, blue) showing enhanced adhesion under 4.45 V stimulation. (f, g, j) Quantification of keratinocyte cytoskeletal and adhesion metrics, exhibiting maximal adhesion and cellular organization at 4.45 V, with diminished responses at supra‐optimal stimulation levels. (k) Cell viability assay (CCK‐8) on Days 1 and 3 shows significantly enhanced metabolic activity of HOKs on SDIA versus Ti control. *Scale bars*, 25 µm. Per‐cell measurements were summarized at the field‐of‐view (FOV) level for statistical analysis. N = 3, mean ± SD. Ns, not significant; ^*^ *p* < 0.05; ^**^ *p* < 0.01; ^***^ *p* < 0.001. One‐way ANOVA with Tukey's post‐hoc test.

Based on the piezoelectric intensity law, we utilized SDIAs polarized for 90 min to attain a piezoelectric intensity range of 3.42 to 5.54 V by controlling the applied force value, so as to determine the optimal piezoelectric intensity for promoting cell adhesion. In order to realistically simulate oral motion, we customized a lateral compression fatigue tester to simulate the process of bioelectrical effects generated by the piezoelectric abutment, whereby the abutment produces lateral piezoelectric charges under vertical stress conditions (Figure 3a). Human gingival fibroblasts (HGFs) and human oral keratinocytes (HOKs) constitute prevalent cell types that play a pivotal role in the process of peri‐implant soft tissue closure [37].

The images presented in Figure 3b showed that the HGFs seeded in the titanium (Ti) group exhibited oval shapes without stretching. The HGFs inoculated onto the SDIA groups initiated a degree of elongation, whereas those cultured on the SDIA group under vertical stress conditions exhibited greater dissemination and more pronounced stretching. In addition, the results showed that the area of adhesion plaques and cell spreading in fibroblasts was higher than that in the Ti and other groups at a bite force value of 100 N (piezoelectric intensity of 4.45 V) (Figure 3b, d, e, h). These findings thus suggested that the electrical signals generated by SDIAs following force application further increased the extension and adhesion of HGFs. Comparable outcomes were attained in the viability assay using live/dead staining (Figure S9). Notably, SDIA had a negligible effect on the viability of HGFs and could promote the proliferation and migration of HGFs, with the strongest effect at 100 N. These findings underscored the safety of bite force‐activated piezo‐catalytic therapy for normal tissues and cells involved in peri‐implantitis healing processes.

To ascertain the cellular biocompatibility of SDIAs, the CCK‐8 assay was used to quantitatively evaluate cell proliferation. As depicted in Figure 3i, the results revealed that the viability of the HGF cells increased following incubation for 1 and 3 days with the SDIA groups compared to that of cells of the Ti group. This finding thus suggested that piezoelectric stimulation was able to promote the proliferation of HGFs. For gingival epithelial cells, they primarily adhere to the abutment surface via hemidesmosomes [38]. Therefore, we evaluated the effects of piezoelectric charges on the adhesion of HOKs by immunofluorescent staining of integrin β4, a major component of hemidesmosomes. The results of the immunofluorescent staining and CCK‐8 live cell counting of HOKs were consistent with those of HGFs, showing the best effect at 100 N. Based on the above results, it can be confirmed that piezoelectric stimulation can promote cell adhesion, proliferation, and migration activities, with the best effect being observed at a voltage value of approximately 4.45 V (Figure 3d‐k  and Figure S9). In addition, we also observed that when the piezoelectric voltage exceeded 4.45 V, the adhesion and migration abilities of cells significantly weakened, indicating that when the piezoelectric intensity exceeds a certain threshold, it may have an adverse effect on cell adhesion. Furthermore, despite numerous studies on charged biomaterials, they primarily focus on utilizing residual polarization charges of materials or high‐frequency piezoelectric charges induced by ultrasound to generate biological effects [39, 40]. Research on low‐frequency stress‐induced piezoelectric charges has only recently been reported [33, 41]. Therefore, the impact of piezoelectric frequency on electrobiological effects remains to be clarified.

ROS exhibit a dual biological role due to fundamental differences in cellular complexity and redox regulation between prokaryotes and eukaryotes [42, 43]. In bacteria, ROS cause irreversible damage to DNA, proteins, and lipids due to limited antioxidant defenses and lack of compartmentalization, making them highly vulnerable to host immune “oxidative bursts” that exploit this weakness for microbial elimination [44, 45]. Conversely, mammalian cells harness low‐to‐moderate ROS levels as regulatory signaling molecules for stimulating proliferation by activating growth pathways (e.g., MAPK/ERK and PI3K/Akt via phosphatase inhibition) and enhancing adhesion through integrin activation and focal adhesion kinase (FAK) signaling. This dichotomy arises because mammalian cells maintain ROS within a tightly regulated “signaling window” using sophisticated antioxidant systems (e.g., glutathione, mitochondrial SOD2) and spatial control, thereby transforming a potential biological hazard into a physiological tool for growth and repair—a key evolutionary adaptation distinguishing simple prokaryotic vulnerability from complex eukaryotic signaling sophistication.

A key concern regarding ROS‐generating materials is their potential to trigger inflammatory responses in host tissues. However, our in vitro results demonstrated that SDIAs did not upregulate pro‐inflammatory cytokines (*IL‐6, TNF‐α*) in macrophages (Figures S10 and S11), effectively alleviating concerns regarding ROS‐induced inflammation. Furthermore, the SDIAs significantly promoted the expression of M2 macrophage markers (*CD206, Arg1*). These thus confirmed that the generated ROS was within a safe and physiological threshold, actively modulating the immune microenvironment toward a pro‐healing and anti‐inflammatory M2 phenotype rather than causing host tissue damage.

### 2.4 In Vitro Antibacterial Properties of SDIAs

To better reflect the real clinical scenarios and further verify the universality and synergistic enhancement effect of SDIAs, ZrO_2_ was specially introduced as the second control group in this study, aiming to systematically compare the comprehensive performance of SDIAs, Ti, and ZrO_2_ in inhibiting bacterial activity.

**FIGURE 4:**
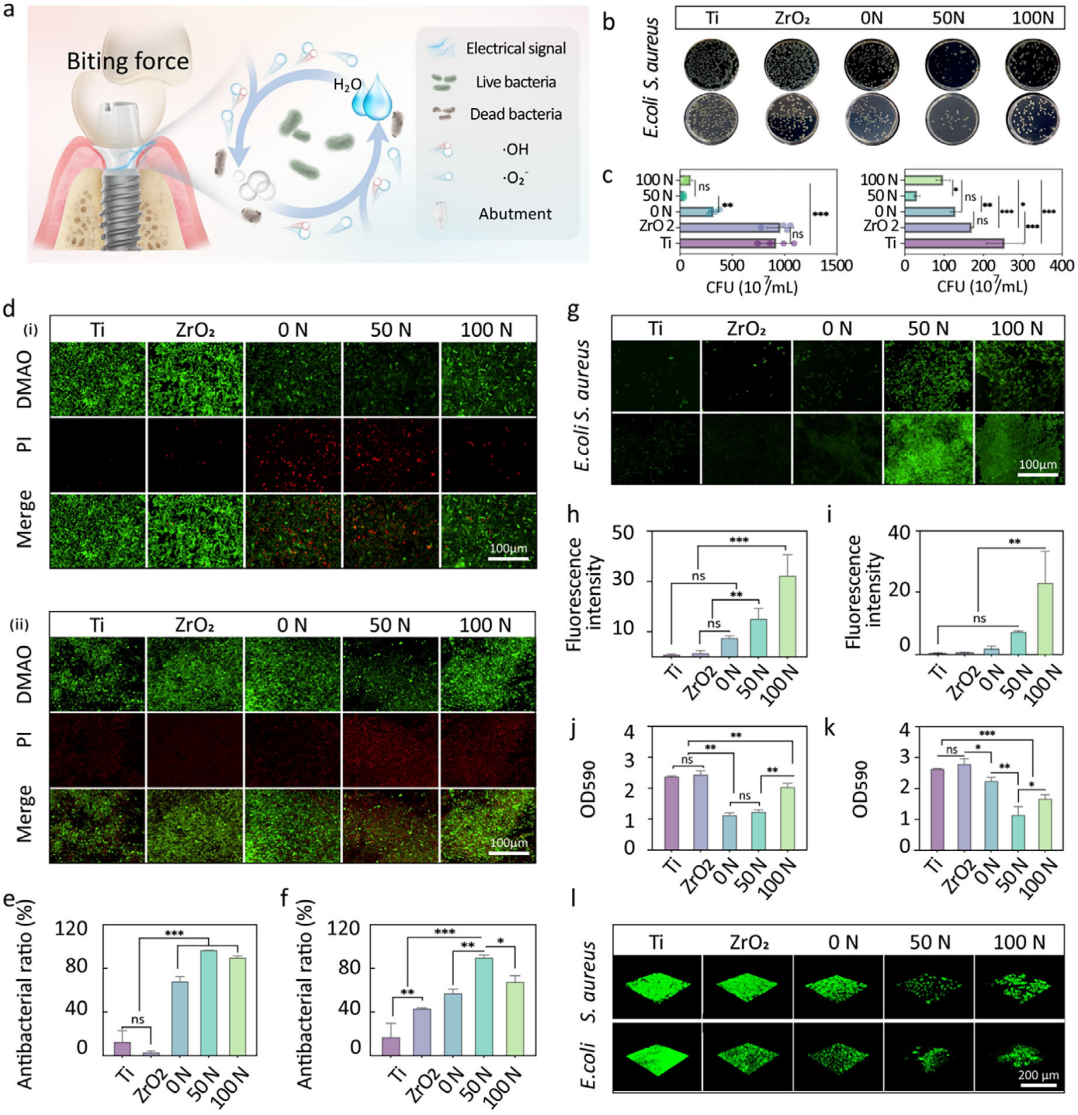
SDIA‐induced bacterial inactivation and biofilm disruption via reactive oxygen species (ROS) production. (a) Schematic illustration of the SDIA piezo‐catalytic antibacterial module showing ROS‐mediated bacterial inactivation under mechanical stimulation. (b, c) Colony‐forming unit (CFU) enumeration by spread plating shows dose‐dependent bacterial reduction for both *Escherichia coli (E. coli)* and *Staphylococcus aureus (S. aureus)* with increasing mechanical force applied to SDIA. (d, e, f) Quantitative analysis of antibacterial efficacy assessed by live/dead staining with DMAPI dyes. Representative data: E. coli demonstrating 67.88% sterilization at 0 N, 96.53% at 50 N, 89.68% at 100 N; S. aureus showing 56.97% at 0 N, 89.68% at 50 N, and 67.74% at 100 N, respectively. (g, h, i) Intracellular oxidative stress measured by DCFH‐DA fluorescence increases with mechanical force amplitude, peaking near 100 N. Quantification of fluorescence intensity confirms significantly elevated ROS levels in SDIA groups compared to Ti and ZrO_2_ controls. Scale bars, 50 µm. (j, k) Biofilm formation following coculture with SDIA was assessed by crystal violet staining assay, demonstrating dose‐dependent reduction in biofilm density with increasing mechanical force. (l) Representative laser‐scanning confocal images of bacterial biofilms visualized by live (green) and dead (red) fluorescent staining with DMAOPI dyes. Scale bars, 100 µm. N = 3, mean ± SD. Ns, not significant; ^*^ *p* < 0.05; ^**^ *p* < 0.01; ^***^ *p* < 0.001. One‐way ANOVA with Tukey's or Kruskal‐Wallis post‐hoc test.

Since soft tissue sealing plays the most crucial role in the anti‐infection effect of the abutment [46], we chose to observe the inhibitory effects of piezoelectricity on bacterial activity within the optimal piezoelectric intensity range that promotes cell adhesion. The pressure force values should not exceed 100 N, and the piezoelectric voltage should not exceed 4.45 V. It was observed that the antibacterial function of SDIAs can not only prevent the invasion of oral microorganisms but can also work in synergy with the soft tissue barrier to prevent infection and enhance the long‐term stability of the implant restoration (Figure 4a). The antibacterial properties of the SDIA were evaluated in vitro against two bacterial strains, *Staphylococcus aureus* (*S. aureus*) and *Escherichia coli* (*E. coli*), which are the typical representative species of Gram‐positive bacteria and Gram‐negative bacteria, respectively. As shown in Figure 4b,c, the bacterial colony‐forming units of *E. coli* and *S. aureus* decreased with increasing bite force on the SDIAs, as assessed by the dilution coating plate method. Additionally, live (Green) / dead (Red) staining of *E. coli* and *S. aureus* was performed and the fluorescent microscopy images showed that the proportions of live bacteria on SDIA decreased, while dead bacteria increased sequentially, with 12.50% (Ti, *E. coli*), 2.74% (ZrO_2,_ *E. coli*), 67.88% (0 N, *E. coli*), 96.53% (50 N, *E. coli*), 89.68% (100 N, *E. coli*), 16.77% (Ti, *S. aureus*), 43.07% (ZrO_2,_ *S. aureus*), 56.97% (0 N, *S. aureus*), 89.68% (50 N, *S. aureus*) and 67.74% (100N, *S. aureus*) sterilization rates (Figure 4d–f).

Since SDIAs generate ROS via piezo‐catalysis and increase the intracellular levels of ROS, which can kill combat pathogenic bacteria [47, 48], we carried out a DCFH‐DA fluorescence staining assay to evaluate the dose‐dependent effects of bite force on intracellular ROS levels within bacteria. As shown in Figure 4g, a gradual increase in ROS level was detected in *E. coli* and *S. aureus* cultured on SDIA with increasing bite force. This was corroborated by quantification of immunofluorescence staining intensities, and it was found that the fluorescence intensities of the SDIA groups were significantly higher compared with the Ti group and ZrO_2_ group, with the SDIA (100 N) group exhibiting the highest value (Figure 4h,i). Additionally, we also evaluated biofilm formation, which is a key mechanism for bacteria to resist harsh environments and enable drug resistance [49, 50]. We detected biofilm formation by live‐dead fluorescence staining and crystal violet staining (Figure 4j–l). The results showed that increasing the bite force on SDIAs led to decreased live rates of *E. coli* and *S. aureus* growing on biofilms, thus validating the antibacterial activity of SDIA (50 N), which displayed the strongest effect. It is worth noting that when the bite force was increased to 100 N, the antibacterial effect of piezoelectricity decreased. Combined with the cell experiment results, it was speculated that this might be related to the cell proliferation‐promoting effect of piezoelectricity [51]. However, compared with the conventional abutment material titanium, SDIA (100 N) still displayed an excellent antibacterial effect and could synergize with piezoelectricity to promote cell adhesion and exert its antibacterial effect. Hence, our study demonstrated that SDIAs elicited dose‐dependent antibacterial effects based on their piezoelectric effect.

### 2.5 SDIAs Promote Cell Adhesion by Regulating the MAPK and PI3K‐Akt Signaling Pathways

**FIGURE 5:**
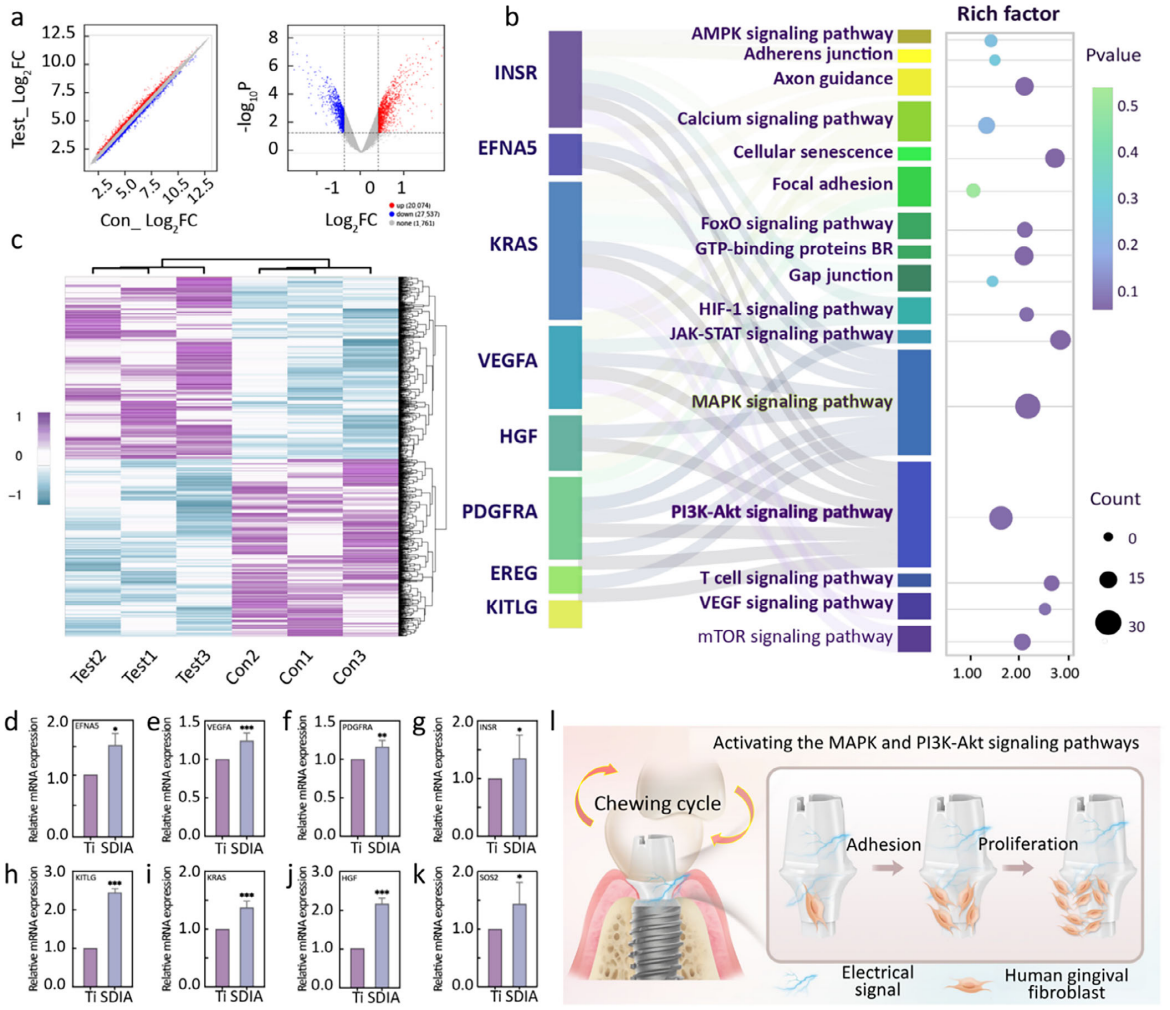
Optimal piezoelectric stimulation activates the MAPK and PI3K‐Akt signaling pathways in human gingival fibroblasts. (a) Sample‐level volcano plot and scatter plot representation of differentially expressed genes (DEGs) in human gingival fibroblasts （HGFs） cultured on SDIA under optimal piezoelectric stimulation (4.45 V) compared to Ti control. Genes above the significance threshold (p < 0.05, |log_2_FC| > 1.5) are highlighted. (b) Pathway enrichment bubble chart showing significantly enriched biological pathways, including mitogen‐activated protein kinase (MAPK) and phosphatidylinositol 3‐kinase (PI3K)‐Akt signaling axes, associated with cell proliferation, migration, and adhesion. (c) Hierarchical clustered heatmap of adhesion‐ and proliferation‐linked genes, demonstrating coordinated upregulation in the SDIA group relative to the Ti control. (d, e, f, g, h, i, j, k) Quantitative reverse transcription‐polymerase chain reaction (RT‐qPCR) validation of selected signaling nodes, including KRAS, INSR, EFNA5, HGF, KITLG, PDGFRA, SOS2, and VEGFA, confirming transcriptomic findings at the mRNA level. (l) Working mechanistic model illustrating that under physiological cyclic loading, SDIA generates piezoelectric potentials that catalyze ROS production to inactivate pathogenic bacteria (E. coli and S. aureus), while simultaneously promoting HGFs adhesion, migration, and proliferation through activation of MAPK and PI3K‐Akt signaling cascades, thereby achieving synchronized cell adhesion and infection control at the peri‐implant interface. Statistics: Differential gene expression analysis was computed using moderated t‐tests with Benjamini‐Hochberg false discovery rate (FDR) correction; p < 0.05 threshold applied. n = 3, mean ± SD. ^*^ *p* < 0.05; ^**^ *p* < 0.01; ^***^ *p* < 0.001. One‐way ANOVA with Tukey's post‐hoc test. Abbreviations: BTO, barium titanate; SDIA, resin‐infiltrated piezoelectric BaTiO_3_; ROS, reactive oxygen species; HGF, human gingival fibroblast; HOK, human oral keratinocyte; VCL, vinculin‐positive focal adhesion.

Having established that SDIAs can significantly better promote cell adhesion and resist bacteria under bite force, we conducted a whole‐genome gene expression analysis on differentially expressed genes of HGFs after optimal electrical stimulation (4.45 V), aiming to identify significantly upregulated signaling pathways in cell expression after electrical stimulation. As expected, differentially expressed genes (DEGs) analysis (Figure 5a) showed that “KRAS” and “INSR” genes [52, 53], known for their key roles in cell proliferation and migration, were highly expressed in HGFs of the SDIA group. The gene enrichment analysis revealed that among the top biological processes highly expressed by HGFs in the SDIA group, there were those related to cell proliferation and migration, such as the MAPK and PI3K‐Akt signaling pathways (Figure 5b). Consistently, cell proliferation and migration‐related gene cluster analysis showed that these genes were significantly enriched in the SDIA group (Figure 5c). These results thus suggested that the HGFs in the SDIA group displayed more obvious transcriptomic characteristics of promoting cell adhesion than those in the Ti group.

To validate the cell adhesion functions of HGFs activated by SDIAs, we further investigated the canonical gene expression in HGFs in response to electrical signals at the mRNA level. The RT‐qPCR results showed that the expression levels of *KRAS*, *INSR*, *EFNA5*, *HGF*, *KITLG*, *PDGFRA*, *SOS2*, and *VEGFA* in HGFs were significantly higher in the SDIA group versus the Ti group (Figure 5d–k). Hence, the above results indicated that the piezoelectric stimulation could enhance the proliferation, migration, and adhesion of HGFs, through activation of the MAPK and PI3K‐Akt signaling pathways (Figure 5l), thereby enhancing their pro‐cell adhesion effects, which in turn promoted subsequent soft tissue integration.

## 3 Conclusion

In summary, we have successfully designed and developed a piezoelectric implant abutment and confirmed the dose‐responsive effects of SDIAs in cell adhesion and combating bacterial infection, thereby preventing the onset of peri‐implantitis. Biocompatibility evaluation showed that SDIAs exhibited minimal cytotoxicity, validating their safety as implant materials. SDIAs significantly enhanced cell proliferation and migration compared with conventional titanium‐based implants. Mechanistically, the SDIAs activated the MAPK and PI3K‐Akt signaling pathways in HGFs to enhance their pro‐cell adhesion functions, thereby enabling them to prevent peri‐implantitis effectively. Antibacterial assays against *E. coli* and *S. aureus* validated the broad‐spectrum, dose‐dependent antibacterial properties of SDIAs under simulated bite force. Further investigations revealed that the piezoelectric stimulation of SDIAs induced ROS production, thereby inhibiting bacterial activity and biofilm formation [54, 55, 56].

## 4 Materials and Methods

### 4.1 Fabrication Procedure for SDIA Piezoelectric Devices

To fabricate BTO ceramic precursors, BTO powders with a particle size of 100 µm were placed in a stainless‐steel mold and compressed into blocks under a pressure of 30 MPa for 25 s. This was followed by the application of an isostatic pressure of 180 MPa for 35 s. Subsequently, the blocks were sintered at temperatures of 1000, 1100, 1200, and 1300°C, with a heating rate of 5°C/min and a holding time of 1 h, before being allowed to cool naturally at temperatures between 20 and 25°C. Prior to polymer infiltration, the BTO porous matrices were treated with a silicone coupling agent to promote chemical bonding between the ceramic and polymer phases of the matrix. The silicone coupling agent used was γ‐MPS (Aldrich Chemical Co., China), which was dissolved in ethanol. A polymer mixture consisting of TEGDMA and Bis‐GMA at a mass ratio of 49.75 wt.% and BPO at 0.5 wt.% was uniformly blended for infiltration. The BTO matrices were immersed in the liquid polymer, facilitating the formation of two interpenetrating phases through capillary action under vacuum conditions. The polymer‐infiltrated BTO matrices were then polymerized via heat treatment at 110°C for 12 h.

### 4.2 Structural Characterization

The phase composition was analyzed via X‐ray diffraction (XRD; Bruker D8 Advance, USA) using Cu‐Kα radiation over 2*θ* = 5°–40° (step size: 0.02°; scan rate: 10°·min^−^^1^). The surface morphology before and after infiltration was characterized using scanning electron microscopy (SEM; Hitachi S4800, Japan). The porosity was quantified by Mercury Intrusion Porosimetry (MIP) using an AutoPore IV 9500 porosimeter (AutoPore IV 9510, USA).

### 4.3 Measurement of Flexural Properties

A nano‐indentation tester (MTS, XP model, USA) was employed to create microcracks in SDIAs for crack observation. A triangular‐pyramidal Berkovich indenter was used with a fixed indentation depth of 500 nm. The flexural strength (σ_F_) and flexural toughness (K_IC_) were determined using three‐point bending and single‐edge V‐notch beam tests per ISO 6872 (dental ceramics) standard, using an Instron 1121 universal tester (Instron, UK). Bars were sectioned to 25 × 3 × 4 mm^3^ (length × width × height). A 1.2‐mm‐deep V‐notch was introduced for fracture toughness measurements. The tests employed a 20‐mm span and 0.5 mm·min^−^^1^ crosshead speed. σ_F_ and K_IC_ were calculated using the following formulas:

$$\;\sigma_{F}=\frac{3Pl}{2wh^{2}}\;\;;\;\;K_{IC}=\frac{P}{w\sqrt{h}}\;\times \frac{l}{h}\times \frac{3\sqrt{\alpha }}{2{\left(1-\alpha \right)}^{1.5}}\times Y$$

where

$\alpha =\frac{a}{w},\;\;Y=1.9109-5.1552\alpha +12.6880\alpha^{2}-19.5736\alpha^{3}+15.9377\alpha^{4}-5.1454\alpha^{5},\;\;P=\;\mathrm{load},\;\;l=\;\mathrm{span},\;\;h=\;\mathrm{height},\;\;w=\;\mathrm{width},\;\;a=\;V-\mathrm{notch}\;\mathrm{depth}\;\left(n\;=5\right)$.

### 4.4 Measurement of Piezoelectric Intensity

The SDIAs were cut into 10 mm ×10 mm ×10 mm blocks for piezoelectric property testing and biological experiments. We conducted corona poling on the sample with an electric field strength of 1 kV mm‐1 for 15, 45, and 90 min to regulate the electromechanical coupling properties of the material blocks. A piezoelectric constant ***d_33_*** tester (Institute of Acoustics of the Chinese Academy of Sciences, China) was used for the *d_33_* measurements. The “quasi‐static” or Berlincourt method was employed. The system works by clamping the sample and subjecting it to a low‐frequency force. Processing the electrical signals from the sample and comparing them with a built‐in reference enabled the system to provide a direct reading of the piezoelectric constant d33. The surface potentials were examined by PeakForce Kelvin Probe Force Microscopy (KPFM‐HV, Bruker, America). To evaluate the direct piezoelectric response under mechanical loading, we subjected the BTO/SDIA blocks to controlled forces using a customized fatigue tester (Xinke Instrumentation Co., Ltd., China). The testing protocol employed 0–200 N cyclic loading at 1.5 Hz frequency to replicate oral motion forces encountered in oral environments, following established methodology from prior studies [57, 58, 59, 60]. Piezoelectric voltages were measured using a Keithley 2450 source meter (Tektronix, USA) when pressure was applied.

### 4.5 Detection of ROS

Hydroxyl radicals (·*OH*) and superoxide anions *(·O_2_^−^)* were quantified using commercial assay kits (Leda Qibo Biotechnology, China). For ·OH quantification, samples were reacted with Reagents 1–3, incubated sequentially (35°C, 1 h; 95°C, 10 min), and absorbance was measured at 532 nm. Net absorbance (***ΔA***) was calculated as ***ATest *** − *** AControl***. For ·*O_2_^−^* analysis, samples containing Reagents 1–3 were incubated at 37°C for 15 min, and the absorbance was measured at 540 nm (***ΔA***  =  ***ATest***  −  ***ABlank***). The concentrations were determined using the following formulas:

$$\mathrm{OH}\;\mathrm{quantification}:\mathrm{Concentration}=\frac{16.7\times \Delta A\times 10^{3}}{W}\;\mathrm{AU}\cdot g^{-1}$$

$$\begin{array}{ccc} & & O_{2}^{-}\;\mathrm{quantification}:\mathrm{Concentration}\\ & & \;=\frac{1075.2\times \left(\Delta A-0.0065\right)}{W}\;\mathrm{nmol}\cdot g^{-1} \end{array}$$

where W = sample mass (g). Quality assurance included preliminary validation studies and adherence to the absorbance threshold criteria.

### 4.6 Cell Culture

*Human gingival fibroblasts* (HGFs, Qincheng Bioscience Inc., Shanghai, China) and *Human oral keratinocytes* (HOKs, ScienCell Research Laboratories, USA) were cultured in Dulbecco's modified Eagle medium (DMEM) supplemented with 10% fetal bovine serum (FBS) and 100 IU mL^−1^ penicillin−streptomycin, at 37°C within a 5% CO_2_ incubator. The medium was changed every 2–3 days. At 80−90% confluence, the cells were detached with 0.25% (*w/v*) trypsin/Ethylenediaminetetraacetic acid (EDTA) (Gibco). Cells from passages 4 to 7 were used in subsequent experiments.

### 4.7 Assessment of Cell Spreading

Cell adhesion structures, F‐actin, hemidesmosomes, and cell nuclei were subject to immunofluorescence staining to observe the adhesion of HGFs and HOKs to the material surface. The cells were seeded into chambers (3 × 10^4^ cells/chamber). After being seeded onto the material surface, the cells in the SDIA group were first subjected to piezoelectric stimulation by applying pressure to the material blocks for 1200 cycles. After piezoelectric stimulation and an additional 6 h of culture, the cells were fixed with 4% (w/v) paraformaldehyde solution and permeabilized with 0.1% (w/v) Triton X‐100. The adhesion structures of HGFs and HOKs were labeled with fluorescent anti‐vinculin primary antibody (1:200, Abcam ab18058, UK) and anti‐integrin β4 primary antibody (1:200, Abcam ab133682, UK), followed by incubation with Alexa Fluor 488 goat anti‐rabbit IgG H&L secondary antibody (1:200, Abcam ab150077, UK), while F‐actin was stained with FITC‐phalloidin. Subsequently, the cells were stained with phalloidin/DAPI (Sigma‐Aldrich, USA) according to the manufacturer's instructions. The stained cells were observed under a laser‐scanning confocal microscope (Olympus 141 FV3000, Tokyo, Japan).

### 4.8 Cell Viability, Proliferation, and Cytotoxicity Assay

For the cell viability assay, cells were seeded into chambers (3 × 10^4^ cells/chamber). Piezoelectric stimulation was applied using 2400 cycles of pressure application on SDIAs. At 1 and 3 days of culture, 10 µL of Cell Counting Kit‐8 (CCK‐8; Bimake, China) was added to 90 µL of cell culture media within each well. After 1 h of incubation, absorbance values were measured using a microplate reader at a wavelength of 450 nm, from which cell viability was computed from a standard curve. For live/dead staining, cells were seeded in chambers (3 × 10^4^ cells/chamber), and piezoelectric stimulation was applied using 2400 cycles of pressure application on the SDIA blocks. After 1 day of culture, the cells were stained with calcein‐AM/PI (Solarbio, China) according to the manufacturer's instructions. The stained cells were observed using a laser‐scanning confocal microscope (Olympus 141 FV3000, Tokyo, Japan).

### 4.9 Total RNA Extraction and Microarray Analysis

Gene expression was analyzed using HGFs exposed to a piezoelectric environment through microarray analysis, with unexposed cells on Ti specimens used as the normal control. Each group had three replicate samples tested, and for each sample, the experiment was performed in triplicate as technical replicates. For Affymetrix microarray profiling, the total RNA was isolated from 1 × 10^6^ cells from the control and treated groups using TRIzol reagent (Invitrogen, Carlsbad, Canada) and purified with an RNeasy Mini Kit (Qiagen, Hilden, Germany) according to the manufacturer's protocol. The amount and quality of RNA were determined using a UV‐Vis spectrophotometer (Thermo, NanoDrop 2000, USA) at an absorbance of 260 nm. The mRNA expression profile was measured using Human Transcriptome Array 2.0 (Affymetrix GeneChip, USA), which contains 44699 gene‐level probe sets. The microarray analysis was performed using Affymetrix Expression Console Software (version 1.2.1). Raw data (CEL files) were normalized at the transcript level using the robust multi‐array average method (RMA workflow). The median summarization of transcript expressions was calculated. The gene‐level data were then filtered to include only those probe sets that were in the ‘core’ meta probe list, which represents RefSeq genes.

### 4.10 Bioinformatics Analysis

For the microarray data analysis, differentially expressed genes were identified based on the random variance model (RVM) *t*‐test. Also, the differentially expressed genes were considered to be up‐ or downregulated with at least p < 0.05. Genes with similar expression patterns often facilitate overlapping functions. Accordingly, cluster analysis of the gene expression patterns was performed using Cluster and Java Treeview software.

### 4.11 Gene Ontology and Pathway Analysis

Gene ontology (GO) analysis was applied to analyze the main function of the differentially expressed genes according to the GO, which can organize genes into hierarchical categories and can uncover the gene regulatory network on the basis of biological processes and molecular function. Specifically, a two‐sided Fisher's exact test and chi squared test were used to classify the GO category, and the false discovery rate (FDR) was calculated to correct the *p*‐value; the smaller the FDR, the smaller the error in judging the *p*‐value.

Pathway analysis was used to determine the significant pathways of the differential genes according to the Kyoto Encyclopedia of Genes and Genomes (KEGG), Biocarta, and Reactome databases. Fisher's exact test was performed to select the statistically significant pathway, and the threshold of significance was considered as p < 0.05.

### 4.12 Quantitative Reverse Transcription Polymerase Chain Reaction (qRT‐PCR)

The expression levels of pathway‐related genes of MAPK and PI3K‐Akt (*KRAS, INSR, EFNAS, HGF, KITLG, PDGFRA, SOS2*, and *VEGFA*) were measured using Quantitative Reverse Transcription Polymerase Chain Reaction (qRT‐PCR). Total RNA was extracted from 1 × 10^6^ cells from the control and piezo‐treated groups using TRIzol reagent (Invitrogen, Carlsbad, Canada) according to the manufacturer's protocol. Equal amounts of total RNA from each sample were reverse transcribed using Thermoscript reverse transcriptase (Invitrogen) with oligo (dT) and random hexamer primers. The primer sequences are presented in Table S1. The qRT‐PCR reaction was performed using a BIORAD instrument with SYBR Green (Takara Bio Inc., Japan), and was run with three biological repeats and three technical repeats. The expression of target genes was normalized to GAPDH (Δ*Ct*) and calculated as fold‐change (2 − ΔΔ*Ct*) vs. controls.

### 4.13 Antibacterial Activity Assay

To assess antibacterial efficacy, *Staphylococcus aureus* (*S. aureus*, ATCC 25923) and *Escherichia coli* (*E. coli*, ATCC 25922) were selected as representative bacteria for efficacy testing. The bacteria were separately grown in LB liquid culture medium at 37°C overnight. The concentration of bacteria was determined by measuring the optical density at 630 nm. The antibacterial effect was evaluated by counting the number of colonies on agar plates. A bacterial suspension (10^5^ CFU mL^−1^, diluted in PBS) was added to the surface of the SDIA and Ti blocks, and the SDIA blocks were subjected to 2400 pressure cycles to activate the piezoelectric charges. Subsequently, the collected specimens were placed in a tube with 1 mL of PBS and vibrated with a vortex device for 60 s to separate the adherent bacteria. The eluent was spread on the LB agar plate, which was then incubated in the incubator for 48 h. Live/dead fluorescent staining of bacteria was performed using the DMAO/PI kit (Beyotime Biotechnology, Shanghai, China) following the manufacturer's instructions and observed using a fluorescence microscope.

The ROS levels were detected using the oxidant‐sensitive dye DCFH‐DA (Reactive Oxygen Species Assay Kit; Beyotime Institute of Biotechnology, China), following the manufacturer's instructions. To evaluate the anti‐biofilm effect of piezoelectric stimulation, a bacterial suspension (10^5^ CFU·mL^−1^, diluted in LB liquid culture medium) was added to the surface of the SDIA and Ti blocks, and the blocks were subjected to 2400 pressure cycles to activate piezoelectric charges. Subsequently, the collected specimens were placed in a tube with 1 mL of PBS and vibrated with a vortex device for 60 s to separate the adhesive bacteria. The bacteria were then cocultured in 12‐well plates for 3 days and subsequently washed gently three times with PBS. Bacterial biofilms were determined by 3D CLSM (SP8; Leica, Germany) using live/dead staining. After washing with PBS, the bacteria were stained with 0.1% (*w/v*) crystal violet solution for 20 min. The stained biofilms were then eluted with a 33% (*v/v*) acetic acid solution, and the absorbance was measured at a wavelength of 595 nm (Spectramax iD5, Molecular Devices, USA).

### 4.14 Statistical Analysis

Statistics for the microarray and bioinformatic analysis were performed by one‐way analysis of variance (ANOVA) using Affymetrix Expression Console TAC (Affymetrix Expression Console), followed by the least significant difference (LSD) test. Statistical differences between groups were analyzed using the independent *t*‐test and one‐way analysis of variance(ANOVA). Data are expressed herein as the mean ± S.D. Significance levels were set at the levels ^*^ *p* < 0.05, ^**^ *p* < 0.01, ^***^ *p* < 0.001 and ^****^ *p* < 0.0001.

## Conflicts of Interest

The authors declare no conflicts of interest.

## Supporting information




**Supporting File**: advs75834‐sup‐0001‐SuppMat.docx.

## Data Availability

The data that support the findings of this study are available from the corresponding author upon reasonable request.

## References

[1] D. Duraccio , F. Mussano , and M. G. Faga , “Biomaterials for Dental Implants: Current and Future Trends,” Journal of Materials Science 50, no. 14 (2015): 4779–4812, 10.1007/s10853-015-9056-3.

[2] D. Buser , L. Sennerby , and H. De Bruyn , “Modern Implant Dentistry Based on Osseointegration: 50 Years of Progress, Current Trends and Open Questions,” Periodontology 73, no. 1 (2017): 7–21, 10.1111/prd.12185. 28000280

[3] K. Gulati and S. Ivanovski , “Dental Implants Modified with Drug Releasing Titania Nanotubes: Therapeutic Potential and Developmental Challenges,” Expert Opinion on Drug Delivery 14, no. 8 (2017): 1009–1024, 10.1080/17425247.2017.1266332. 27892717

[4] T. Guo , K. Gulati , H. Arora , P. Han , B. Fournier , and S. Ivanovski , “Race to Invade: Understanding Soft Tissue Integration at the Transmucosal Region of Titanium Dental Implants,” Dental Materials 37, no. 5 (2021): 816–831, 10.1016/j.dental.2021.02.005. 33676764

[5] T. Guo , K. Gulati , H. Arora , P. Han , B. Fournier , and S. Ivanovski , “Orchestrating Soft Tissue Integration at the Transmucosal Region of Titanium Implants,” Acta Biomaterialia 124 (2021): 33–49, 10.1016/j.actbio.2021.01.001. 33444803

[6] P.‐I. Branemark , “Osseointegration and Its Experimental Background,” The Journal of Prosthetic Dentistry 50, no. 3 (1983): 399–410, 10.1016/S0022-3913(83)80101-2. 6352924

[7] S. Ivanovski and R. Lee , “Comparison of Peri‐Implant and Periodontal Marginal Soft Tissues in Health and Disease,” Periodontology 76, no. 1 (2018): 116–130, 10.1111/prd.12150. 29193334

[8] S. Li , W. Zhao , Y. Huang , et al., “A Sericin@Cu Coating on Zirconia to Orchestrate a Sequential Antibacterial‐to‐Regenerative Cascade for Enhancing Peri‐Implant Soft Tissue Seal,” Advanced Healthcare Material 15 (2026): 05161, 10.1002/adhm.202505161. 41518263

[9] G. Shineh , L. M. Janghour , Y. Xia , et al., “Biomolecule‐functionalized Dental Implant Surfaces: towards Augmenting Soft Tissue Integration,” Bioactive Materials 53 (2025): 540–590, 10.1016/j.bioactmat.2025.07.005. 40755849 PMC12313982

[10] Z. Wang , P. Tuerxun , T. Ng , et al., “Enhancing Angiogenesis in Peri‐implant Soft Tissue with Bioactive Silk Fibroin Microgroove Coatings on Zirconia Surfaces,” Regener Biomater 11 (2024): rbae068, 10.1093/rb/rbae068. PMC11257716 39027360

[11] S. Wang , Z. Wu , Y. Wang , et al., “A Homogeneous Dopamine–silver Nanocomposite Coating: Striking a Balance between the Antibacterial Ability and Cytocompatibility of Dental Implants,” Regener Biomater 10 (2023): rbac082, 10.1093/rb/rbac082. PMC9847628 36683759

[12] Y. Yuan , M. Zhou , J. Yu , M. Chen , J. Kang , and H. Wei , “The “Barrier‐erecting” of Titanium Dental Implant: Surface Modification Strategies for Enhancing Soft Tissue Integration,” Biomaterials 326 (2026): 123697, 10.1016/j.biomaterials.2025.123697. 40966911

[13] S. Wu , J. Xu , L. Zou , et al., “Long‐lasting Renewable Antibacterial Porous Polymeric Coatings Enable Titanium Biomaterials to Prevent and Treat Peri‐implant Infection,” Nature Communications 12, no. 1 (2021): 3303, 10.1038/s41467-021-23069-0. PMC8175680 34083518

[14] M.‐E. Jennes , M. Naumann , S. Peroz , F. Beuer , and F. Schmidt , “Antibacterial Effects of Modified Implant Abutment Surfaces for the Prevention of Peri‐Implantitis—A Systematic Review,” Antibiotics 10, no. 11 (2021): 1350, 10.3390/antibiotics10111350. 34827288 PMC8615005

[15] X. Lu , Z. Wu , K. Xu , et al., “Multifunctional Coatings of Titanium Implants toward Promoting Osseointegration and Preventing Infection: Recent Developments,” Frontiers in Bioengineering and Biotechnology 9 (2021): 783816, 10.3389/fbioe.2021.783816. 34950645 PMC8691702

[16] P. M. Maquera‐Huacho , G. G. de Carvalho , M. Jafelicci Júnior , E. Marcantonio Junior , and D. M. P. Spolidorio , “Physical‐chemical Influences and Cell Behavior of Natural Compounds on Titanium Dental Surfaces,” Brazilian Dental Journal 34: 553–562, 10.1590/0103-6440202305582. PMC10759949 38133473

[17] H. J. Haugen , S. Makhtari , S. Ahmadi , and B. Hussain , “The Antibacterial and Cytotoxic Effects of Silver Nanoparticles Coated Titanium Implants: a Narrative Review,” Materials 15, no. 14 (2022): 5025, 10.3390/ma15145025. 35888492 PMC9320431

[18] A. V. Zanfir , G. Voicu , C. Busuioc , S. I. Jinga , M. G. Albu , and F. Iordache , “New Coll–HA/BT Composite Materials for Hard Tissue Engineering,” Materials Science and Engineering: C 62 (2016): 795–805, 10.1016/j.msec.2016.02.041. 26952486

[19] M. M. Vijatovic , J. D. Bobic , and B. D. Stojanovic , “History and Challenges of Barium Titanate: Part I,” Science of Sintering 40, no. 2 (2008): 155–165, 10.2298/SOS0802155V.

[20] J. Chen , W. Li , L. Zhou , et al., “A Built‐in Electric Field with Nanoscale Distinction for Cell Behavior Regulation,” Journal of Materials Chemistry B 6, no. 18 (2018): 2723–2727, 10.1039/C8TB00063H. 32254224

[21] A. S. Motamedi , H. Mirzadeh , F. Hajiesmaeilbaigi , S. Bagheri‐Khoulenjani , and M. A. Shokrgozar , “Piezoelectric Electrospun Nanocomposite Comprising Au NPs/PVDF for Nerve Tissue Engineering,” Journal of Biomedical Materials Research Part A 105, no. 7 (2017): 1984–1993, 10.1002/jbm.a.36050. 28256789

[22] T. Yao , J. Chen , Z. Wang , et al., “The Antibacterial Effect of Potassium‐sodium Niobate Ceramics Based on Controlling Piezoelectric Properties,” Colloids and Surfaces B: Biointerfaces 175 (2019): 463–468, 10.1016/j.colsurfb.2018.12.022. 30572154

[23] A. Dhall , S. Islam , M. Park , Y. Zhang , A. Kim , and G. Hwang , “Bimodal Nanocomposite Platform with Antibiofilm and Self‐Powering Functionalities for Biomedical Applications,” ACS Applied Materials & Interfaces 13, no. 34 (2021): 40379–40391, 10.1021/acsami.1c11791. 34406755 PMC8548987

[24] W. Han , Z. Liu , H. Yu , et al., “An Artificial Piezoelectric‐Conductive Integrated Peri‐Implant Gingiva Enables Efficient Bacterial Inhibition and Soft‐Tissue Integration,” Advanced Fiber Materials 7, no. 4 (2025): 1128–1147, 10.1007/s42765-025-00543-8.

[25] X. Wu , S. Sheng , Y. Xu , J. Shi , X. Ning , and A. Zhou , “Electric Eel‐Inspired Bioelectromechanical Bandage with Biochemical‐Photothermal‐Piezoelectric Synergy for Promoting Postoperative Recovery in Diabetes,” ACS Nano 19, no. 30 (2025): 27665–27691, 10.1021/acsnano.5c07405. 40705023

[26] R. Luo , Y. Xiong , J. Li , et al., “Piezoelectric Injectable Anti‐Adhesive Hydrogel to Promote Endogenous Healing of Tendon Injuries,” Advanced Materials 37 (2025): 2501306, 10.1002/adma.202501306. 40658817

[27] Q. Xu , X. Gao , S. Zhao , et al., “Construction of Bio‐Piezoelectric Platforms: from Structures and Synthesis to Applications,” Advanced Materials 33, no. 27 (2021): 2008452, 10.1002/adma.202008452. 34033180 PMC11469329

[28] Y. Zang , F. Zhang , C. Di , and D. Zhu , “Advances of Flexible Pressure Sensors Toward Artificial Intelligence and Health Care Applications,” Materials Horizons 2, no. 2 (2015): 140–156, 10.1039/C4MH00147H.

[29] J. Li , X.‐H. Zhang , B.‐C. Cui , et al., “Mechanical Performance of Polymer‐infiltrated Zirconia Ceramics,” Journal of Dentistry 58 (2017): 60–66, 10.1016/j.jdent.2017.01.008. 28159508

[30] H. Wang , B. Cui , J. Li , et al., “Mechanical Properties and Biocompatibility of Polymer Infiltrated Sodium Aluminum Silicate Restorative Composites,” Journal of Advanced Ceramics 6, no. 1 (2017): 73–79, 10.1007/s40145-016-0214-0.

[31] P. A. Mataga , “Deformation of Crack‐bridging Ductile Reinforcements in Toughened Brittle Materials,” Acta Metallurgica 37, no. 12 (1989): 3349–3359, 10.1016/0001-6160(89)90207-1.

[32] B. Gang and H. Chung‐Yuen , “Effects of Interface Debonding on the Toughness of Ductile‐particle Reinforced Ceramics,” International Journal of Solids and Structures 26, no. 5–6 (1990): 631–642, 10.1016/0020-7683(90)90034-S.

[33] J. Li , X. Zhao , Y. Xia , et al., “Strontium‐Containing Piezoelectric Biofilm Promotes Dentin Tissue Regeneration,” Advanced Materials 36, no. 21 (2024): 2313419, 10.1002/adma.202313419. 38335452

[34] H. Huang , S. Tu , C. Zeng , T. Zhang , A. H. Reshak , and Y. Zhang , “Macroscopic Polarization Enhancement Promoting Photo‐ and Piezoelectric‐Induced Charge Separation and Molecular Oxygen Activation,” Angewandte Chemie International Edition 56, no. 39 (2017): 11860–11864, 10.1002/anie.201706549. 28731229

[35] J. M. C. Po , J. A. Kieser , L. M. Gallo , A. J. Tésenyi , P. Herbison , and M. Farella , “Time‐Frequency Analysis of Chewing Activity in the Natural Environment,” Journal of Dental Research 90, no. 10 (2011): 1206–1210, 10.1177/0022034511416669. 21810620

[36] F. A. Fontijn‐Tekamp , A. P. Slagter , A. Van Der Bilt , et al., “Biting and Chewing in Overdentures, Full Dentures, and Natural Dentitions,” Journal of Dental Research 79, no. 7 (2000): 1519–1524, 10.1177/00220345000790071501. 11005738

[37] X. Ren , H. C. van der Mei , Y. Ren , and H. J. Busscher , “Keratinocytes Protect Soft‐tissue Integration of Dental Implant Materials against Bacterial Challenges in a 3D‐tissue Infection Model,” Acta Biomaterialia 96 (2019): 237–246, 10.1016/j.actbio.2019.07.015. 31302293

[38] X. Miao , D. Wang , L. Xu , et al., “The Response of human Osteoblasts, Epithelial Cells, Fibroblasts, Macrophages and Oral Bacteria to Nanostructured Titanium Surfaces: a Systematic Study,” International Journal of Nanomedicine 12 (2017): 1415–1430, 10.2147/IJN.S126760. 28260888 PMC5325133

[39] M. Wu , Z. Zhang , Z. Liu , et al., “Piezoelectric Nanocomposites for Sonodynamic Bacterial Elimination and Wound Healing,” Nano Today 37 (2021): 101104, 10.1016/j.nantod.2021.101104.

[40] Y. Bai , X. Zheng , X. Zhong , et al., “Manipulation of Heterogeneous Surface Electric Potential Promotes Osteogenesis by Strengthening RGD Peptide Binding and Cellular Mechanosensing,” Advanced Materials 35, no. 24 (2023): 2209769, 10.1002/adma.202209769. 36934418

[41] T. Li , C. Shi , F. Jin , et al., “Cell Activity Modulation and Its Specific Function Maintenance by Bioinspired Electromechanical Nanogenerator,” Science Advances 7, no. 39 (2021): abh2350, 10.1126/sciadv.abh2350. PMC8462902 34559554

[42] J. Sun , J. Zhang , H. Lei , et al., “An ‘anaerobic Invisible’ genetically Engineered Bacterium for Periodontitis Treatment through Temporal Modulation on Microenvironment,” Dental Research 1, no. 1 (2026): 100002, 10.1016/j.dtrs.2025.100002.

[43] Y. Lu , Y. Guo , X. Guo , et al., “Two‐dimensional FeCu‐metal‐organic‐framework Boosts Peroxidase Activity for Antibacterial Application,” Dental Research 1, no. 1 (2026): 100008, 10.1016/j.dtrs.2025.100008.

[44] D. Choudhary , V. Lagage , K. R. Foster , and S. Uphoff , “Phenotypic Heterogeneity in the Bacterial Oxidative Stress Response Is Driven by Cell‐cell Interactions,” Cell Reports 42, no. 3 (2023): 112168, 10.1016/j.celrep.2023.112168. 36848288 PMC10935545

[45] Y. Li , S. Hao , Y. Fan , et al., “Development of Berberine Derivative‐loaded Nanovesicles for ROS‐driven Eradication of Helicobacter pylori: Overcoming Antibiotic Resistance and Disruption of Gut Microbiota,” Nano Research 18, no. 8 (2025): 94907674, 10.26599/NR.2025.94907674.

[46] C. W. Hall and T.‐F. Mah , “Molecular Mechanisms of Biofilm‐based Antibiotic Resistance and Tolerance in Pathogenic Bacteria,” FEMS Microbiology Reviews 41, no. 3 (2017): 276–301, 10.1093/femsre/fux010. 28369412

[47] B. Jia , S. Zhang , L. Zhang , et al., “Unveiling the Role of Intermittent Electrostimulation: Enhancing Microbial Metabolism and Electron Transfer in Electroactive Biofilms to Optimize V(V) Reduction and Immobilization,” ACS ES&T Engineering 5, no. 6 (2025): 1538–1550, 10.1021/acsestengg.5c00005.

[48] G. Zimmermann , B. Papke , S. Ismail , et al., “Small Molecule Inhibition of the KRAS–PDEδ Interaction Impairs Oncogenic KRAS Signalling,” Nature 497, no. 7451 (2013): 638–642, 10.1038/nature12205. 23698361

[49] C. M. Taniguchi , B. Emanuelli , and C. R. Kahn , “Critical Nodes in Signalling Pathways: Insights into Insulin Action,” Nature Reviews Molecular Cell Biology 7, no. 2 (2006): 85–96, 10.1038/nrm1837. 16493415

[50] R. Alexander and X. Liu , “Soft Tissue Integration around Dental Implants: a Pressing Priority,” Biomaterials 324 (2026): 123491, 10.1016/j.biomaterials.2025.123491. 40505390 PMC12226153

[51] B. Ezraty , A. Gennaris , F. Barras , and J.‐F. Collet , “Oxidative Stress, Protein Damage and Repair in Bacteria,” Nature Reviews Microbiology 15, no. 7 (2017): 385–396, 10.1038/nrmicro.2017.26. 28420885

[52] X. Wu , M. Yang , J. S. Kim , et al., “Reactivity Differences Enable ROS for Selective Ablation of Bacteria,” Angewandte Chemie International Edition 61, no. 17 (2022): 202200808, 10.1002/anie.202200808. 35174598

[53] J. Yan and B. L. Bassler , “Surviving as a Community: Antibiotic Tolerance and Persistence in Bacterial Biofilms,” Cell Host & Microbe 26, no. 1 (2019): 15–21, 10.1016/j.chom.2019.06.002. 31295420 PMC6629468

[54] A. I. Mohammed , S. Sangha , H. Nguyen , et al., “Assessment of Oxidative Stress‐Induced Oral Epithelial Toxicity,” Biomolecules 13, no. 8 (2023): 1239, 10.3390/biom13081239. 37627304 PMC10452318

[55] Y. S. Raval , L. Flurin , A. Mohamed , K. E. Greenwood‐Quaintance , H. Beyenal , and R. Patel , “In Vitro Antibacterial Activity of Hydrogen Peroxide and Hypochlorous Acid, Including That Generated by Electrochemical Scaffolds,” Antimicrobial Agents and Chemotherapy (2021), e01966, 10.1128/AAC.01966-20. 33649112 PMC8092879

[56] G. E. Iliakis , G. E. Pantelias , R. Okayasu , and W. F. Blakely , “Induction by H_2_O_2_ of DNA and Interphase Chromosome Damage in Plateau‐Phase Chinese Hamster Ovary Cells,” Radiation Research 131, no. 2 (1992): 192, 10.2307/3578441. 1641473

[57] C. H. Gibbs , P. E. Mahan , H. C. Lundeen , K. Brehnan , E. K. Walsh , and W. B. Holbrook , “Occlusal Forces during Chewing and Swallowing as Measured by Sound Transmission,” The Journal of Prosthetic Dentistry 46, no. 4 (1981): 443–449, 10.1016/0022-3913(81)90455-8. 6946215

[58] D. J. Anderson , “Measurement of Stress in Mastication. I,” Journal of Dental Research 35, no. 5 (1956): 664–670, 10.1177/00220345560350050201. 13367282

[59] H. M. Edmonds and H. Glowacka , “The Ontogeny of Maximum Bite Force in Humans,” Journal of Anatomy 237, no. 3 (2020): 529–542, 10.1111/joa.13218. 32406523 PMC7476206

[60] K. Kohyama , E. Hatakeyama , T. Sasaki , H. Dan , T. Azuma , and K. Karita , “Effects of Sample Hardness on human Chewing Force: a Model Study Using Silicone Rubber,” Archives of Oral Biology 49, no. 10 (2004): 805–816, 10.1016/j.archoralbio.2004.04.006. 15308425

