# Supplementary material for: Oral Motion‐Powered Smart Dental Implant Abutment for In Situ Antibacterial and Cell Adhesion Through Piezoelectric Effect
Source: Adv Sci (Weinh). 2026 May 26:e23944. Online ahead of print. doi: 10.1002/advs.202523944 (PMC13336118; doi:10.1002/advs.202523944)
Supplement: Supplementary file 1 — Supporting file: advs75834‐sup‐0001‐SuppMat.docx [file ADVS-9999-e23944-s001.docx]

**Supporting Information**

**Oral Motion-Powered Smart Dental Implant Abutment for In Situ Antibacterial and Cell Adhesion through Piezoelectric Effect**

Authors: *Xiyu Shi, Xiaoyu Han, Yanhui Lu, Yuan Chai, Boon Chin Heng, Baiyan Xiao, Shuo Liu, Tingting Wu, Tingjun Li, Qiaomei Ren, Ting Song, Le Chen, Dong Han***, Yaru Guo***, Xuliang Deng***, Xuehui Zhang**

**TABLE S1 |** Primer Sequences Used for Quantitative Reverse Transcription Polymerase Chain Reaction(qRT-PCR) Analysis.

| Primers | Forward Sequence (5'to3') | Reverse Sequence (5'to3') |
| --- | --- | --- |
| AREG | TGTCGCTCTTGATACTCGGC | ATGGTTCACGCTTCCCAGAG |
| EFNA5 | TAACCGGCCTCACTCTCCAA | GGACCTTCTTCCATTATCTGGGA |
| EREG | GGAGGATGGAGATGCTCTGTG | ACTGAGGACTGCCTGTAGAAGA |
| HGF | AGCAAGAAAACAATGCCTCTGG | GCTGCGTCCTTTACCAATGA |
| INSR | AGACGTCCCGTCAAATATTGC | CCATCTGGCTGCCTCTTTCT |
| KITLG | GCCAGCTCCCTTAGGAATGAC | GACTTGGCTGTCTCTTCTTCC |
| KRAS | TGGGGAGGGCTTTCTTTGTGTA | GGACCATAGGTACATCTTCAGAGTC |
| PDGFRA | AAAGTGGAGGAGACCATCGC | ATCACCAACAGCACCAGGAC |
| SOS2 | GCCTTACGAGTTCTTCAGCG | CTTGTTCCTGAACCTTCCGC |
| VEGFA | GAGCCTTGCCTTGCTGCTCTA | CACCAGGGTCTCGATTGGATG |

**Figure S1**


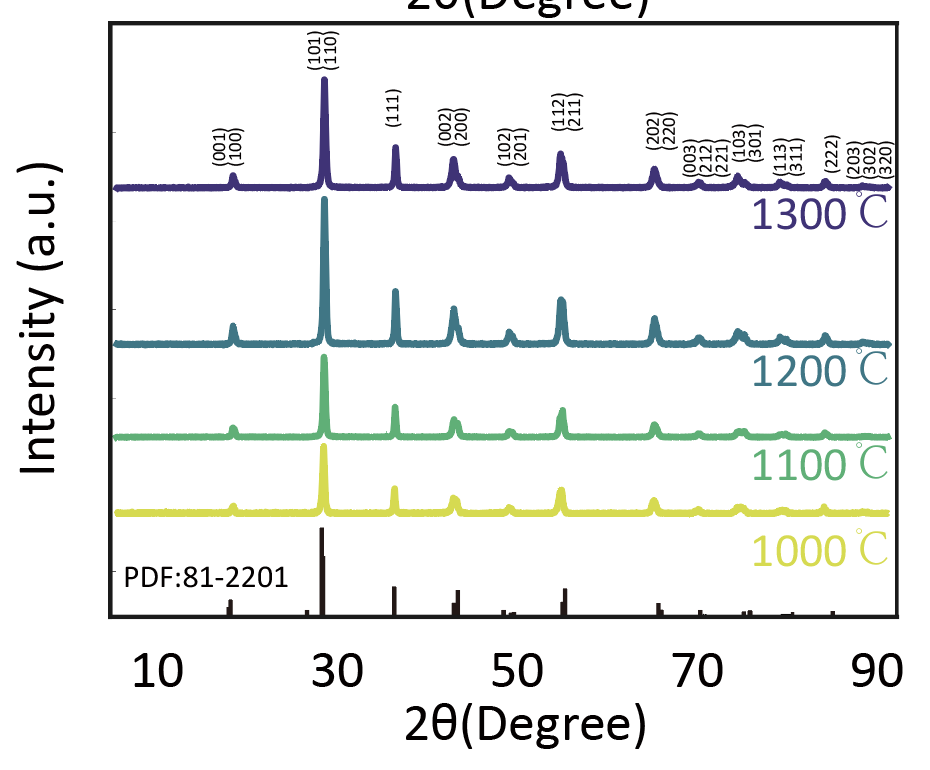


**FIGURE S1 |** XRD spectrum of BTO ceramics sintered by different temperature.

**Figure S2**


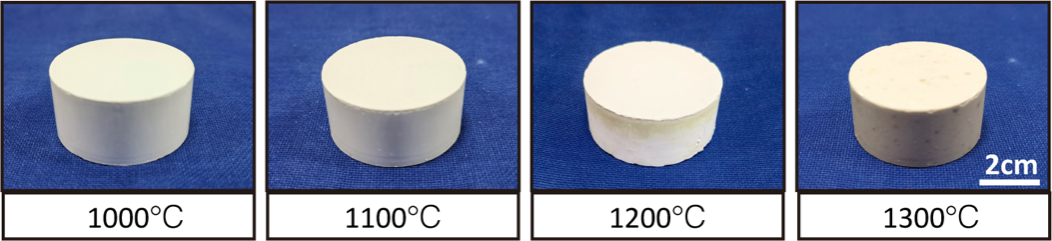


**FIGURE S2 |** BTO ceramics samples before toughness reinforced sintered.

**Figure S3**


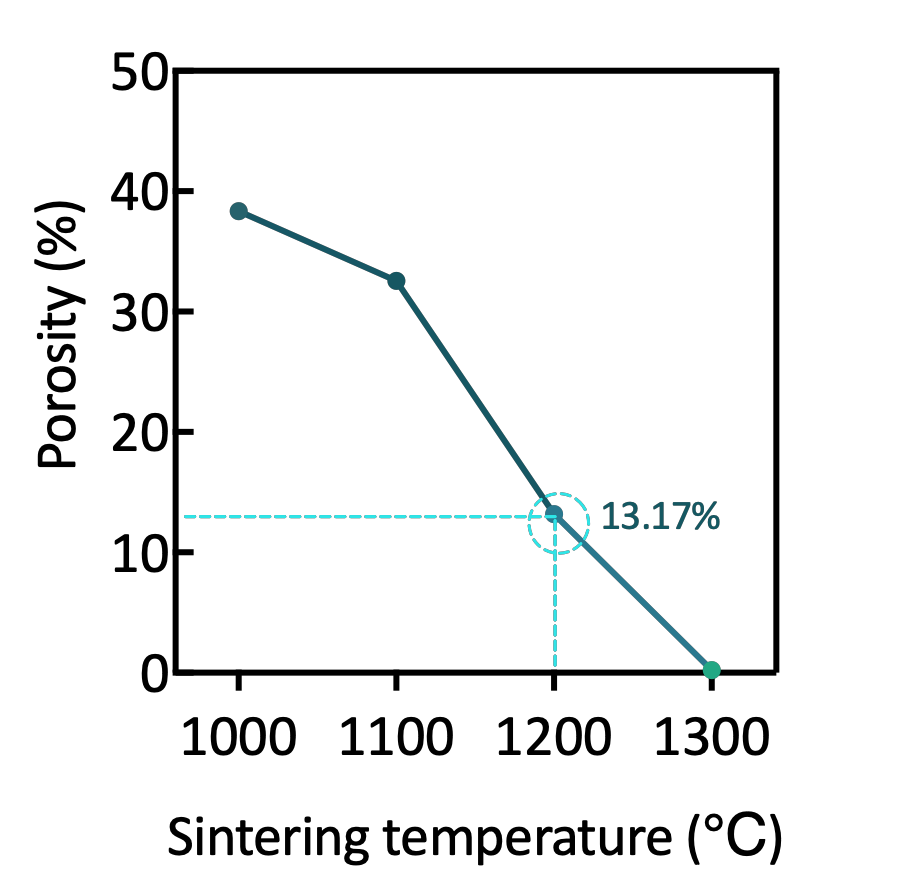


**FIGURE S3 |** Porosity of BTO ceramics sintered by different temperature.

**Figure S4**


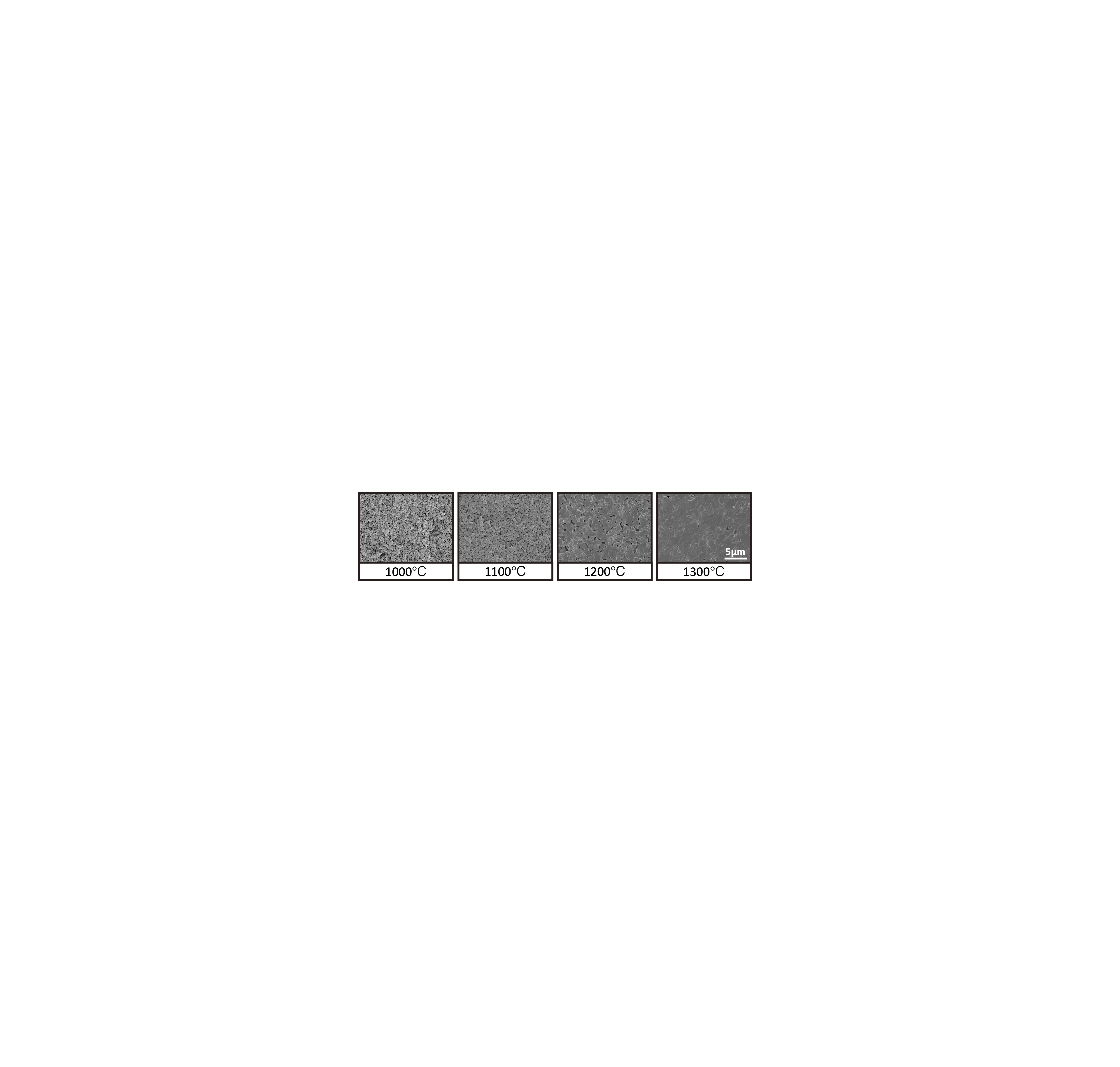


**FIGURE S4 |** SEM observation of BTO ceramics samples before toughness reinforced.

**Figure S5**


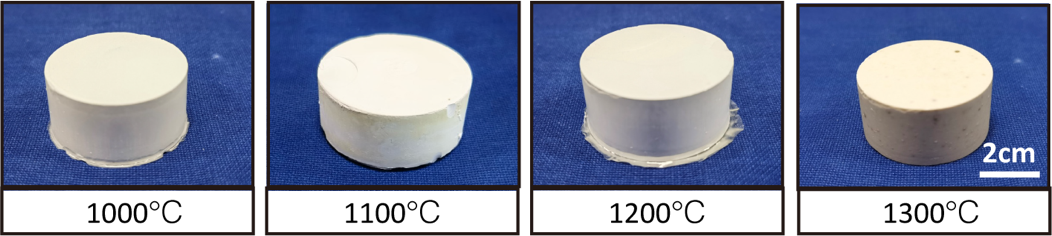


**FIGURE S5 |** BTO ceramics samples after toughness reinforced sintered.

**Figure S6**


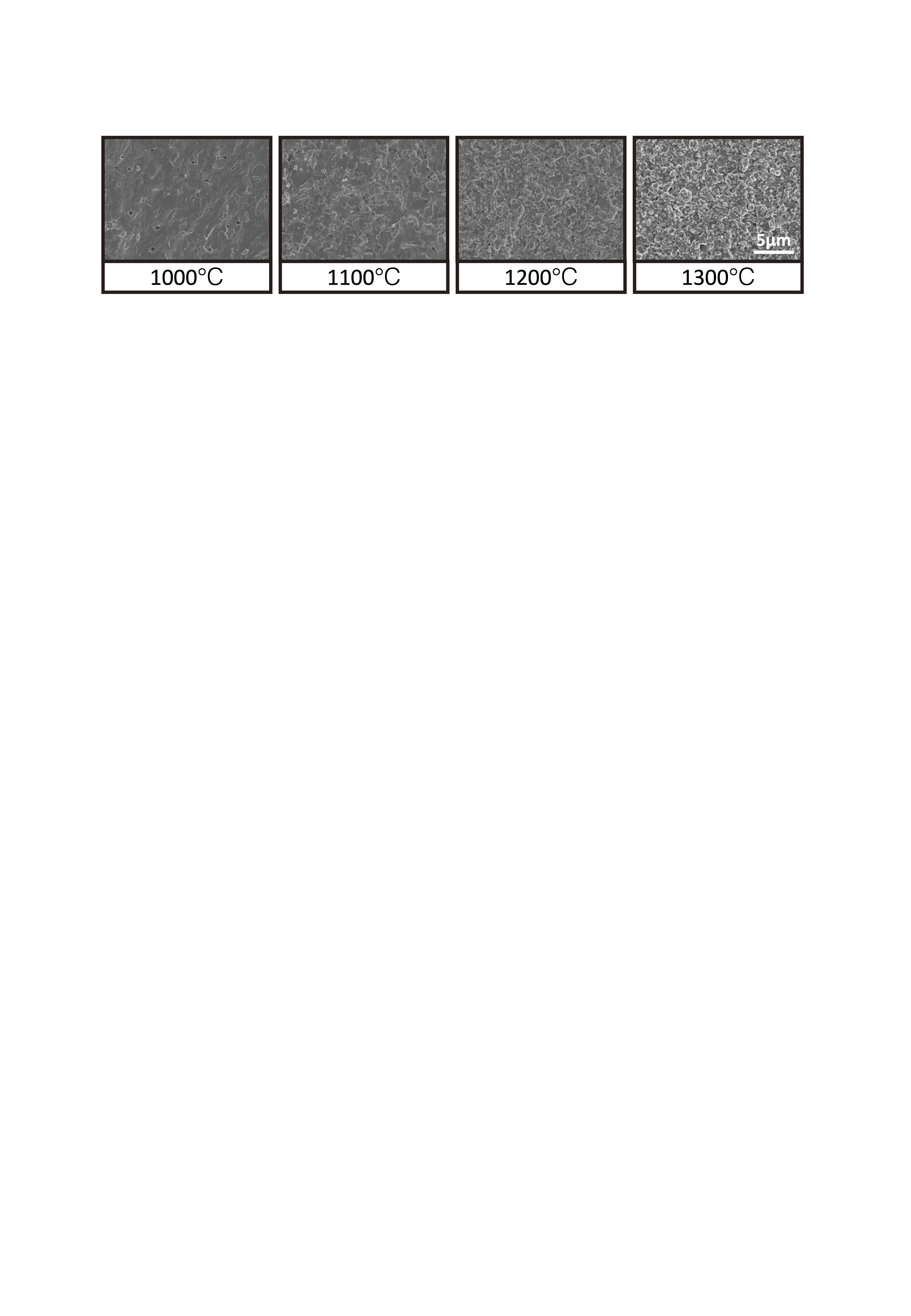


**FIGURE S6 |** SEM observation of BTO ceramics samples after toughness reinforced.

**Figure S7**


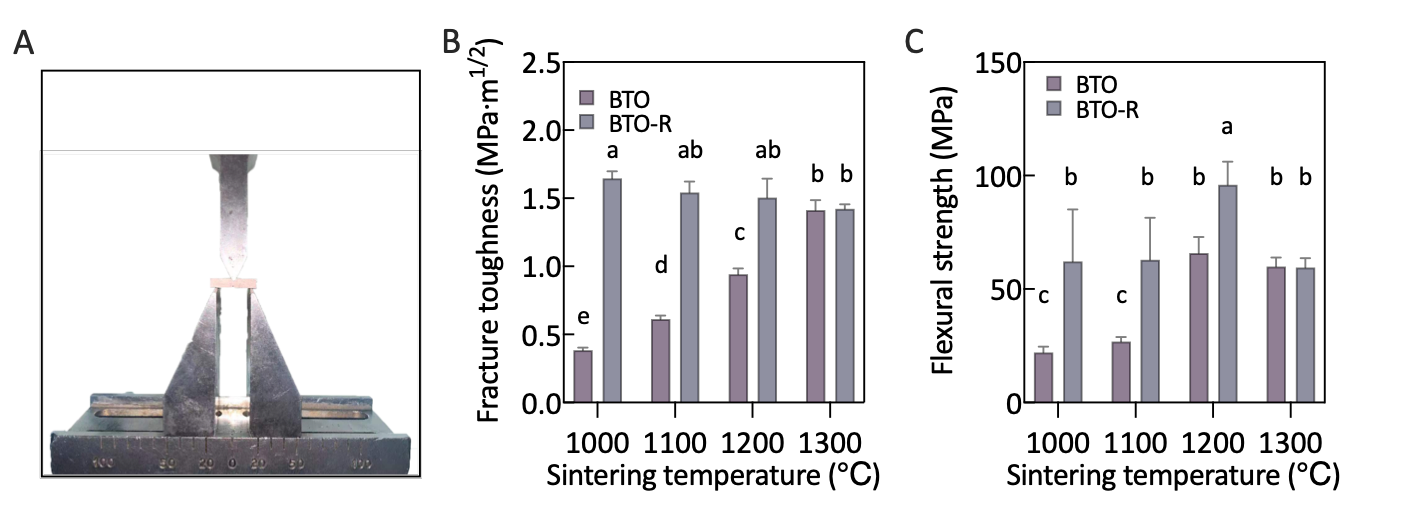


**FIGURE S7 |** Mechanical property test of BTO ceramics. (A) Picture of three point bending test. (B-C) Flexural strength and fracture toughness results of BTO ceramics before and after toughness reinforcement at different sintering temperatures. (n = 4, mean ± s.e.m., **P* = 0.0217, ***P* = 0.040, ****P* < 0.001, *****P* < 0.0001)

**Figure S8**


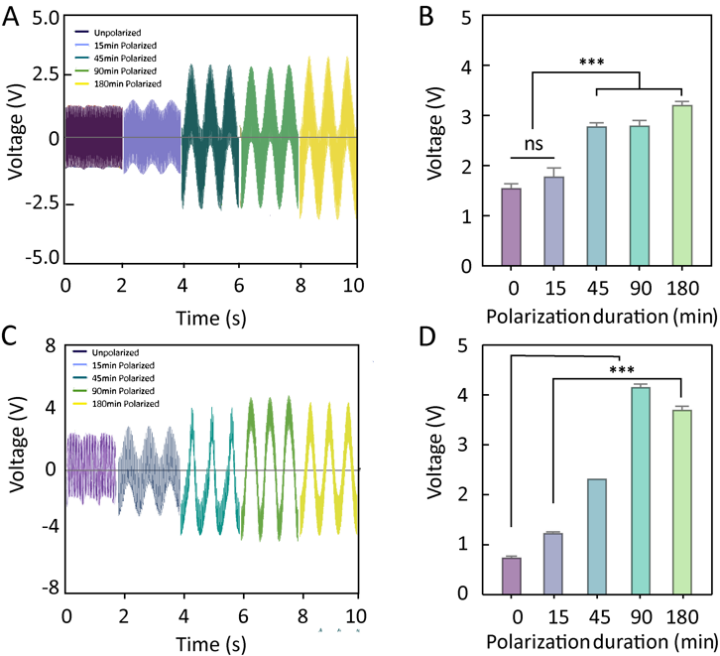


**FIGURE S8 |** Optimized polarization effects in reinforced BTO Ceramics under mechanical Loading**.** (A)Voltage-time profiles of BTO-R samples under 70N biting force, showing waveform evolution across polarization durations (0/15/45/90/180 min). (B) Comparative peak voltages at 70N loading (n = 3, mean ± SD., **p* < 0.05, ***p* < 0.01, ****p* < 0.001, *****p* < 0.0001). (C)Voltage transients under 200N biting force, exhibiting amplified signal amplitude and sharper waveforms. (D) Maximum output voltages (200N) confirming performance saturation at 90-min polarization (n = 3, mean ± SD., **p* < 0.05, ***p* < 0.01, ****p* < 0.001, *****p* < 0.0001).

**Figure S9**


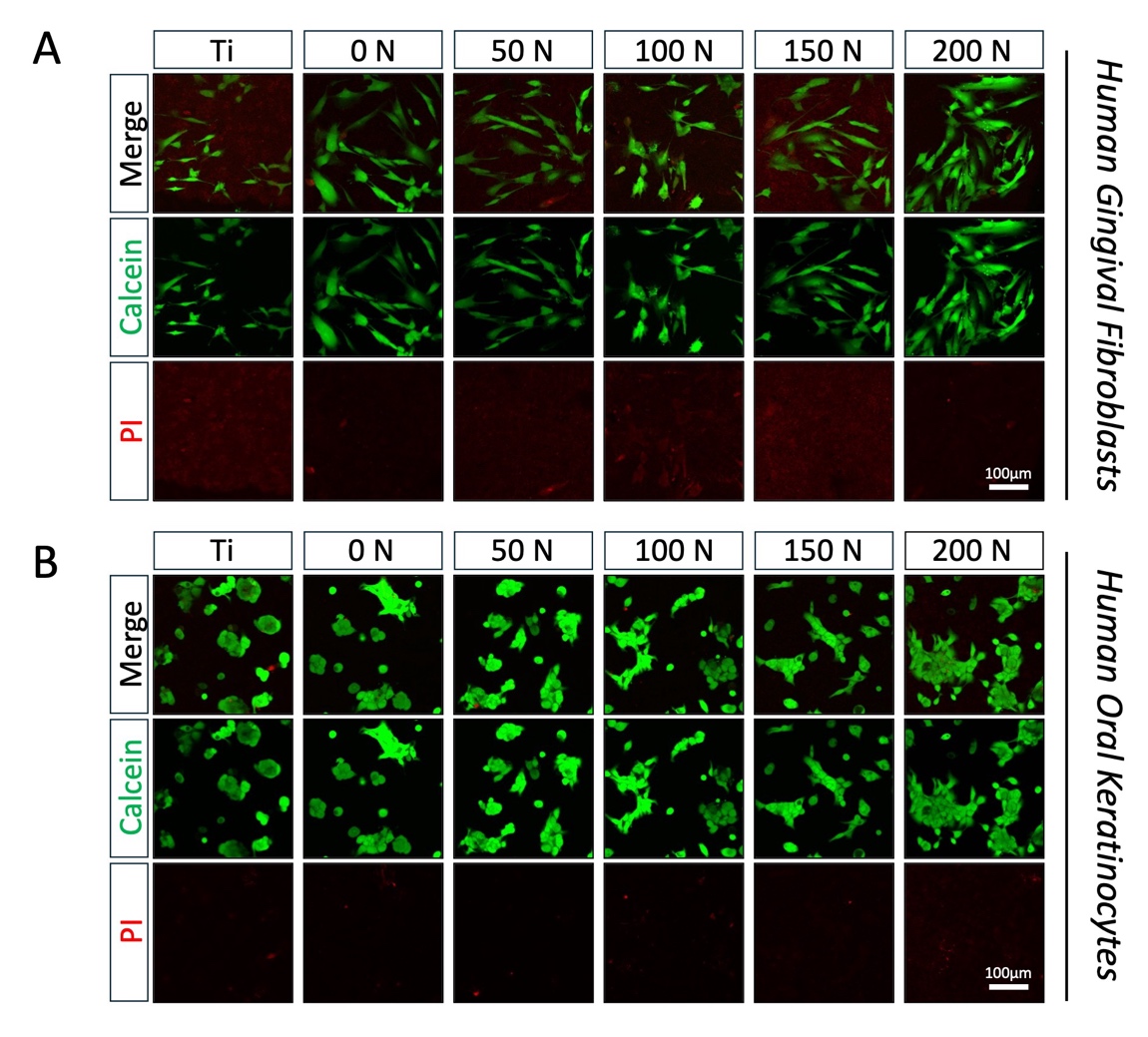


**FIGURE S9 |** Live/Dead staining images of A) Human Gingival Fibroblasts (HGFs) and B) Human Oral Keratinocytes (HOKs) after different piezoelectric stimulations. All materials were subjected to 2400 loading cycles.

**Figure S10**


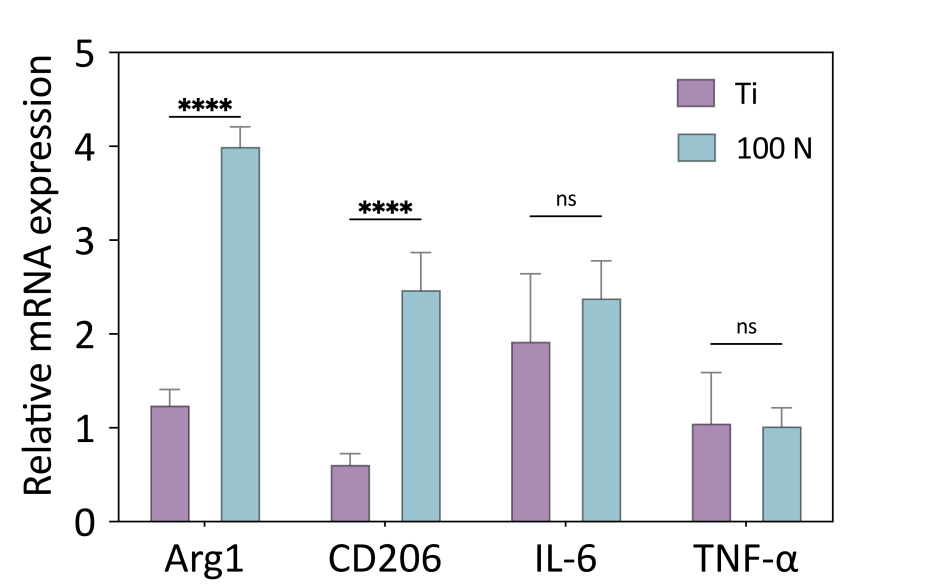


**FIGURE S10 |** Quantitative gene expression analysis of macrophages cultured on the sample surfaces. Relative mRNA expression levels of M1-related pro-inflammatory genes (*IL-6, TNF-α*) and M2-related anti-inflammatory/pro-healing genes (*Arg1*, *CD206*) in RAW 264.7 macrophages, determined by RT-qPCR. Data are presented as mean ± SD (n = 3). ****p < 0.001*, *ns* = not significantly different compared to the Control Ti group

**Figure S11**

**FIGURE S11 |** Evaluation of macrophage polarization on different sample surfaces by immunofluorescence staining. Representative immunostaining images of RAW 264.7 macrophages cultured on Control Ti and SDIA surfaces. The cells were fluorescently stained for the M2 macrophage marker CD206 (green) and nuclear DNA (DAPI, blue). Scale bar: 100 μm.
